# Rational design of a live-attenuated eastern equine encephalitis virus vaccine through informed mutation of virulence determinants

**DOI:** 10.1371/journal.ppat.1007584

**Published:** 2019-02-11

**Authors:** Derek W. Trobaugh, Chengqun Sun, Matthew D. Dunn, Douglas S. Reed, William B. Klimstra

**Affiliations:** Center for Vaccine Research, Department of Immunology, University of Pittsburgh, Pittsburgh, PA United States of America; University of Colorado Denver, UNITED STATES

## Abstract

Live attenuated vaccines (LAVs), if sufficiently safe, provide the most potent and durable anti-pathogen responses in vaccinees with single immunizations commonly yielding lifelong immunity. Historically, viral LAVs were derived by blind passage of virulent strains in cultured cells resulting in adaptation to culture and a loss of fitness and disease-causing potential *in vivo*. Mutations associated with these phenomena have been identified but rarely have specific attenuation mechanisms been ascribed, thereby limiting understanding of the attenuating characteristics of the LAV strain and applicability of the attenuation mechanism to other vaccines. Furthermore, the attenuated phenotype is often associated with single nucleotide changes in the viral genome, which can easily revert to the virulent sequence during replication in animals. Here, we have used a rational approach to attenuation of eastern equine encephalitis virus (EEEV), a mosquito-transmitted alphavirus that is among the most acutely human-virulent viruses endemic to North America and has potential for use as an aerosolized bioweapon. Currently, there is no licensed antiviral therapy or vaccine for this virus. Four virulence loci in the EEEV genome were identified and were mutated individually and in combination to abrogate virulence and to resist reversion. The resultant viruses were tested for virulence in mice to examine the degree of attenuation and efficacy was tested by subcutaneous or aerosol challenge with wild type EEEV. Importantly, all viruses containing three or more mutations were avirulent after intracerebral infection of mice, indicating a very high degree of attenuation. All vaccines protected from subcutaneous EEEV challenge while a single vaccine with three mutations provided reproducible, near-complete protection against aerosol challenge. These results suggest that informed mutation of virulence determinants is a productive strategy for production of LAVs even with highly virulent viruses such as EEEV. Furthermore, these results can be directly applied to mutation of analogous virulence loci to create LAVs from other viruses.

## Author summary

Live-attenuated vaccines (LAVs) mimic a natural virus infection and elicit high levels of neutralizing antibodies that can persist for long times. Historically, LAVs have been created by blind passaging of the virus leading to attenuating mutations in the viral genome with no known mechanism of action. We have used an informed approach to create a LAV for eastern equine encephalitis virus (EEEV). EEEV is one of the most highly virulent mosquito-borne viruses in the United States, and there is currently no approved vaccine or antiviral therapeutic. Here, we created a series of LAVs by combining mutations of four alphavirus virulence loci that have known functions. We demonstrate that viruses containing at last three mutations are highly attenuated after both a subcutaneous and intracerebral infection of mice and provide protective immunity against both a subcutaneous and aerosol challenge. We have also identified a key mutation, elimination of the miR-142-3p microRNA biding sites in the EEEV 3’ untranslated region, as critical for myeloid cell replication and essential for eliciting optimal cytokine responses, T cell responses, and protection from challenge. In summary, our results provide a rationale for an informed approach to the generation of LAVs against arboviruses.

## Introduction

Vaccines against virus pathogens have been licensed in the United States since 1914 [1] and most are inactivated or live-attenuated viruses. Inactivated vaccines are viruses that have been killed using either formaldehyde (polio virus, influenza virus, and hepatitis A virus) or β-propiolactone (influenza virus) rendering the virus unable to replicate after vaccination [2]. Live-attenuated vaccines (LAVs) are live viruses that either have been mutated, most commonly by blind passage (e.g., [3]), or exhibit host incompatibility to reduce virulence after vaccination [4]. Smallpox, measles, mumps and rubella virus (MMR), varicella virus (chicken pox), rotavirus, and yellow fever virus vaccines are LAVs that are currently FDA approved [5,6].

LAVs have an advantage over inactivated vaccines in their ability to mimic natural virus infection, thus inducing a potent immune response that results in high levels of neutralizing antibodies that persist for longer times as well as inducing T cell responses to epitopes scattered throughout the virus genome [7]. One LAV in particular, the yellow fever virus (YFV) 17D vaccine, induces a neutralizing antibody response in >95% of vaccinees that can persist for >35 years [8]. The YFV 17D vaccine was generated by blind serial passaging of a virulent YFV strain in mouse and chicken tissues [3] leading to the accumulation of 31 amino acid mutations in comparison with the Asibi parental strain [9]. Similar blind serial passaging has been used to generate other LAVs including the oral poliovirus vaccine [10], the Venezuelan equine encephalitis virus (VEEV) TC83 vaccine [11], the chikungunya virus (CHIKV) 181/25 vaccine [12] and the Japanese encephalitis vaccine SA14-14-2 [13]. While effective in attenuating virulent viruses, serial passaging introduces mutations in the virus genome that have unknown mechanisms of action and can exhibit minimal genetic differences compared to virulent parental strains [5]. For example, the LAV poliovirus vaccine strain contains 10 essential attenuating mutations [14] and reversion of these mutations yielding virulent viruses can occur rapidly in vaccinees [15] putting unimmunized populations at risk. LAVs are often contraindicated in young or immunocompromised populations because of safety concerns [16].

Inactivated vaccines are inherently safer than LAVs, but often, they require formulation with adjuvants and multiple booster immunizations to achieve protective antibody responses. These antibody responses can wane over time, leading to inadequate protection and reemergence of the pathogen [17]. Inactivation techniques (e.g. formalin) can also disrupt the natural epitopes on the surface of virus particles changing the antibody repertoire [18]. Furthermore, inactivated vaccines may not effectively stimulate the adaptive immune response to generate memory T cell responses [19,20]. A rationally-designed LAV would preserve the natural epitopes of the virus while also effectively stimulating both the humoral and adaptive immune response; yet be sufficiently attenuated for administration to most populations and resistant to reversion.

The mosquito-borne alphaviruses are members of the *Togaviridae* family of medically-important viruses that cause encephalitis (EEEV, VEEV, and western (WEEV) equine encephalitis) or arthritis and arthralgia (e.g., CHIKV, Sindbis virus, and Ross River virus) [21]. EEEV is endemic in the Eastern US and is among the most virulent acutely infectious viruses known, resulting in a 30–70% mortality rate in symptomatic cases and long-term neurological sequelae in most surviving humans [22,23]. Currently, there are no licensed antivirals or an approved vaccine for any of the alphaviruses. A formalin-inactivated EEEV vaccine that is given to at risk workers was developed by the United States Army in the 1960s and remains under investigational new drug status [24,25]. However, the vaccine is poorly immunogenic and requires repeated booster immunizations to maintain adequate serum neutralizing antibody levels [24]. An inactivated EEEV/WEEV vaccine is available for veterinary use, but this also requires multiple booster shots in endemic areas [26].

For an EEEV LAV to be licensed, two main outcomes would need to be achieved: 1) adequate virus attenuation to prevent potential adverse events with this highly virulent virus [27], and 2) sufficient virus replication for induction of highly protective immunity. To begin to design an EEEV LAV, we chose four target loci for inclusion, mutations at each of which had been shown to affect either EEEV virulence or the virulence of other encephalitic alphaviruses in animal models. These included: 1) A locus in the 5’ untranslated region (UTR) that was originally identified in the VEEV blind passage TC-83 LAV that alters the secondary structure of the UTR compared to wild-type (WT) VEEV strains and increases the binding and translation suppression of IFIT-1, an interferon-induced antiviral effector protein [28]. 2) A five amino acid deletion of a nuclear localization signal in the capsid protein that reduces shutoff of host cell transcription [29–32]. 3) A three amino acid charged-alanine change in the E2 glycoprotein that greatly reduces heparan sulfate (HS) binding by the virus [33,34]. 4) Deletion of the four miR-142-3p microRNA binding sites in the EEEV 3’ UTR that leads to efficient EEEV infection of myeloid cells and induction of virus-attenuating systemic interferon-α/β (IFN-α/β) [35].

We designed LAV candidates containing mutations in each of the loci, singly or in combination, creating a series of LAV candidates. Mutations were designed such that reversion to WT phenotypes would require more than a single nucleotide change as is often the case with LAVs derived through blind passage [3,10–12]. The LAVs were screened for defects in virus growth *in vitro*, attenuation in a mouse model of EEEV pathogenesis and protection against high dose subcutaneous (sc) or aerosol EEEV challenge. LAVs with mutations in three or four virulence loci were fully attenuated in a mouse model and conferred complete protection against sc challenge and partial or complete protection against high dose aerosol challenge. Critically, the vaccine viruses containing three or more mutations were completely avirulent from an intracerebral inoculation route, a contrast with multiple LAVs derived from blind passage (e.g., YFV 17D and VEEV TC83) and suggesting a large margin of safety. Together, our data demonstrates that rational design of LAVs through mutation of known virulence loci can be effective tool in generating LAVs against alphaviruses and potentially other RNA virus pathogens.

## Results

### Construction of the EEEV LAV candidates and virus growth kinetics in Vero cells

To generate the LAV candidates, we disrupted four virulence loci in the wild-type (WT) EEEV strain, FL93-939. We specifically designed the mutations to resist reversion by mutating either multiple nucleotides, multiple amino acids or creating a deletion; thus, at least two nucleotide changes would be required for reversion (Table 1). First, to increase sensitivity to IFIT-1, nucleotides 4 and 6 in the 5’ UTR were mutated from guanine to adenine (5’U4&6) to disrupt the 5’ terminal stem-loop structure similar to the VEEV TC-83 LAV 5’ UTR [28]. Second, to eliminate shut-off of host transcription, amino acids 65–69 comprising a nuclear localization sequence, were deleted from the capsid protein (C65-69) [32]. Third, three lysine residues at amino acids 71, 74, and 77 in the E2 protein were mutated to alanine to disrupt binding of virus particle to cell surface heparan sulfate (HS) (E71-77) increasing virus replication in lymphoid tissues and significantly reducing virus spread within the CNS [33,34]. Finally, 260 nucleotides were deleted in the 3’ UTR to remove the miR-142-3p binding sites to allow for efficient replication in myeloid cells (3’U11337) and the induction of systemic IFN-α/β [35]. The mutations were incorporated individually or in combination into the EEEV cDNA clone to generate the 14 LAV candidates (Table 1). Virus stocks of the single mutants were sequenced and confirmed the presence of the desired mutation within the rescued virus stock. A triple mutant virus possessing the WT EEEV 5’UTR was omitted due to a desire to test triple and quadruple mutation viruses that encode a 5’UTR mutation. This mutation is critical for attenuation of the VEEV TC83 vaccine strain [11,28], an investigational new drug vaccine candidate that is given to at risk-laboratory workers [36].

**Table 1:** WT EEEV LAV candidates to be tested for virulence and immunogenicity.

Mutation	Virus	Mutations	Predicted effect	Viable virus	Titer (PFU/ml)	Genomic equivalents (GE/ml)	Specific Infectivity (GE/PFU)
	WT EEEV FL93-939	-	-	+	1.0x10^10^	2.16x10^12^	216
1	5’U4&6	G-A at nucleotide 4 and 6 of 5’ UTR	Increases IFIT1 sensitivity	+	1.x10^10^	7.08x10^11^	71
2	C65-69	Delete capsid aa 65–69 nuclear translocation signal	Reduces transcriptional shutoff	+	1.78x10^10^	1.18x10^12^	106
3	E71-77	Lys-Ala at E2 71, 74, 77	HS binding-negative, increases lymphoid tissue targeting	+	1.48x10^8^	1.65x10^12^	11,786
4	3’U11337	Delete residues 11337–11596 of 3’ UTR	Eliminate mir142-3p sensitivity, increases myeloid cell replication/decreases mosquito infectivity	+	1.56x10^9^	8.9x10^11^	570
1, 2	5’U4&6 C65-69			+	9.5x10^8^	1.43x10^11^	151
1, 3	5’U4&6 E71-77			+	9.8x10^6^	1.37x10^11^	13,979
1, 4	5’U4&6 3’U11337			+	1.58x10^9^	9.58x10^11^	606
2, 3	C65-69 E71-77			+	6.5x10^7^	1.01x10^12^	15,538
2, 4	C65-69 3’U11337			+	3.5x10^9^	3.97x10^11^	113
3, 4	E71-77 3’U11337			+	1.58x10^7^	6.78x10^11^	42,911
1, 2, 3	5’U4&6 C65-69 E71-77			+	2.2x10^7^	4.05x10^11^	18,409
1, 2, 4	5’U4&6 C65-69 3’U11337			+	1.18x10^9^	5.17x10^11^	438
1, 3, 4	5’U4&6 E71-77 3’U11337			+	1.18x10^7^	3.0x10^11^	25,424
1, 2, 3, 4	5’U4&6 C65-69 E71-77 3’U11337			+	2.0x10^7^	1.25x10^12^	62,500

**Fig 1:**
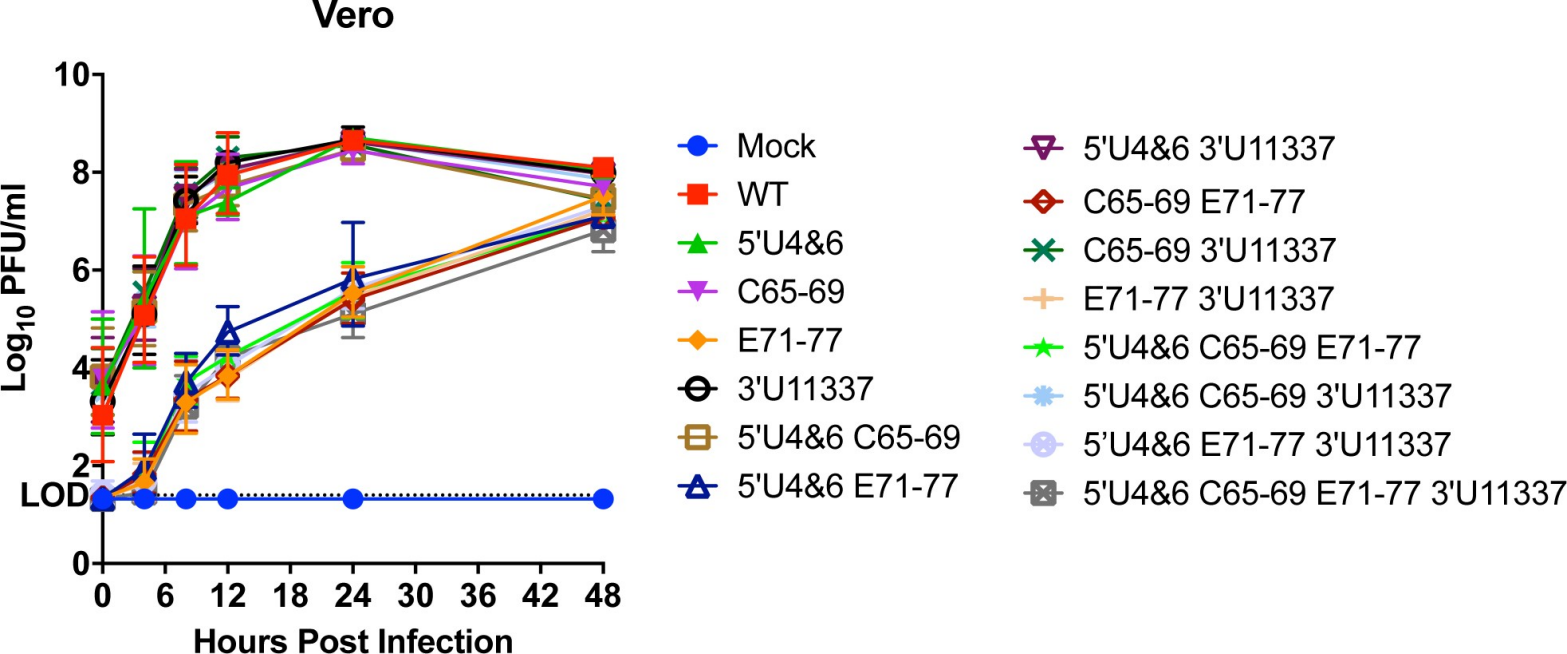
LAV replication *in vitro* in Vero cells. Vero cells were infected with equal genomes of the LAV candidates corresponding to a multiplicity of infection equal to 1 PFU per cell for WT EEEV. Data is represented as geometric mean and error bars representing standard deviation of each data point. Each data point is from 2 independent experiments that were performed in triplicate. LOD = limit of detection.

To determine whether incorporation of the mutations into the EEEV cDNA clone affected virus growth *in vitro*, Vero cells were infected with equal genomes of each of the LAV vaccine candidates to compensate for differences in the specific infectivity (genome equivalents [GE] to PFU ratio) of the HS-binding and non-HS binding mutants (Table 1) [33,37,38]. Genome numbers were calculated based on the number of genomes equivalent to a MOI = 1 for WT EEEV. The growth kinetics of the LAV candidates were separated into two main groups: those that should bind efficiently to HS and those that contained the E71-77 mutation and presumably lacked the ability to bind to HS (Fig 1). Similar to previously published results, the HS binding-deficient mutants exhibited slower growth kinetics compared to the HS-binding LAV candidates due to inefficient binding of virus particles to the cell surface [33,34]. At 12 hpi, there was 10,000-fold increase in virus replication of the HS-binding LAVs compared to the non-HS-binding LAVs (Fig 1). By 48 hours, this difference was eliminated as yields became similar between the HS binding and the non-HS biding (E71-77) viruses most likely due to eventual infection of all cells by the non-HS binding viruses. Importantly, incorporation of any of the four mutations singly or in combination into the LAVs had no detrimental effect on virus growth in this context. Vero cells do not express miR-142-3p [35], therefore differential effects of the presence of the miR-142-3p binding sites should not be evident. These results demonstrate that all of the LAVs are viable, have similar growth kinetics *in vitro*, and can be considered for potential further testing *in vivo*.

### Increasing the number of mutations leads to greater virus attenuation after primary infection of mice

**Fig 2:**
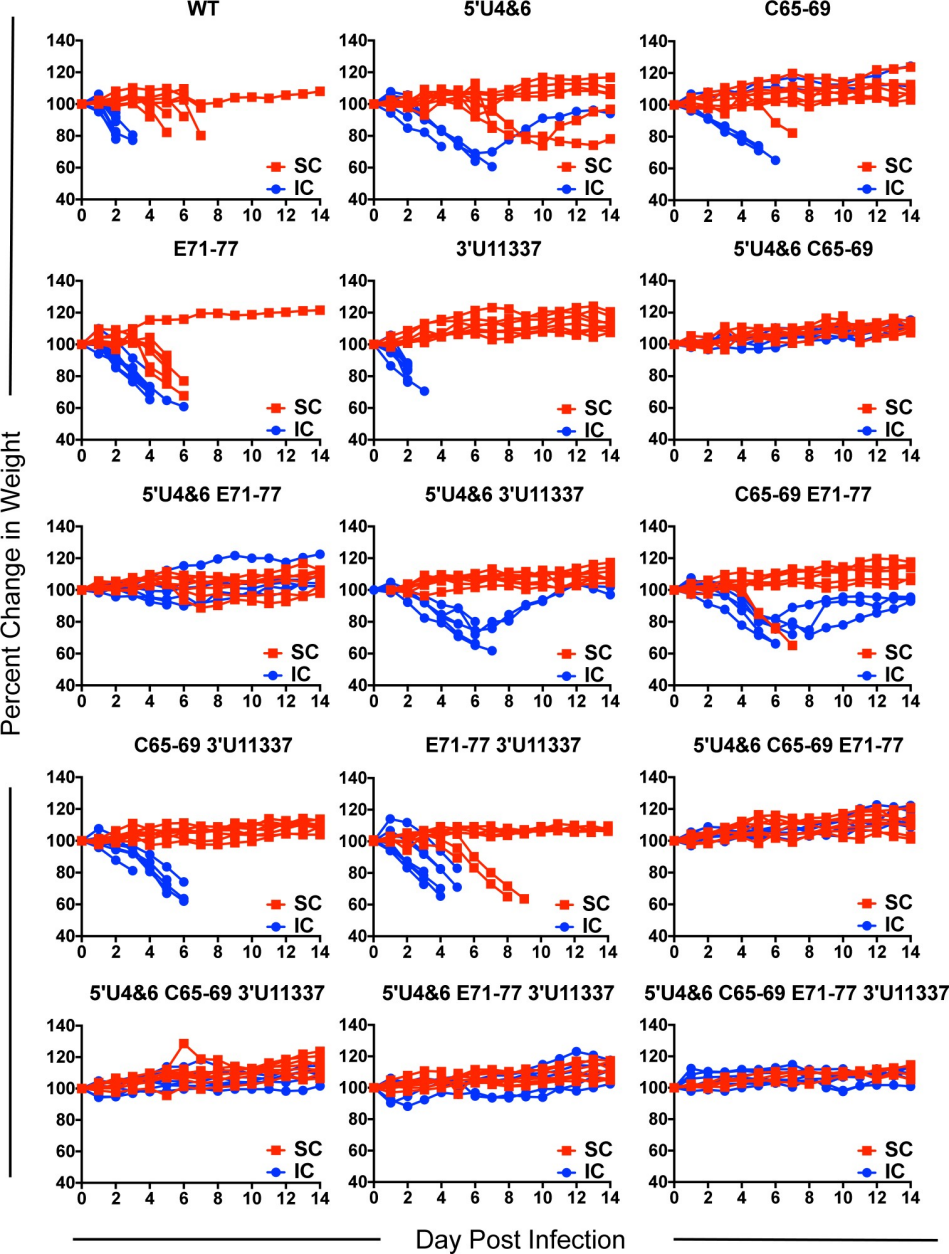
Mouse weight loss after subcutaneous and intracerebral LAV infection. CD-1 mice were infected with equal genomes of the LAV candidates corresponding to 10^3^ pfu of WT EEEV in both rear footpads. Percent change in weight was calculated based on mouse weights on day 0 of experiment. Each line represents a single mouse. n = 6–8 mice) from 2 independent experiments for either subcutaneous (SC, red) or intracerebral (IC, blue) infection.

To begin to examine the ability of the LAV candidates to function as attenuated and immunogenic vaccine vectors *in vivo*, we examined virulence of the single mutants as well as all combinations of the mutations following a primary subcutaneous (sc) or intracerebral (ic) infection (Table 2). Female outbred CD-1 mice (6 weeks old) were infected with equal genomes of the viruses corresponding to 10^3^ pfu (1.5 x 10^5^ genomic equivalents) of WT EEEV subcutaneously (sc), in each rear footpad or intracerebrally (ic), and monitored daily for morbidity and mortality. Following sc infection, the WT EEEV and single mutation E71-77 virus resulted in limited survival (8.3% and 4.2%,respectively) with a small increased mean time to death (MTD) for the E71-77 mutant (Table 2), similar to previously reported results [33]. The 3’U11337 LAV candidate was significantly attenuated compared to WT (mutant 91.3% survival; P<0.0001, Log-Rank Test). Survival of the 3’U11337 virus was higher in these experiments than previously published results [35] potentially due to use of the outbred CD-1 model. Infection with both 5’U4&6 (survival 45.8%; P<0.0001) and C65-69 (survival 91.7%; P<0.0001) single mutants also resulted in significant attenuation compared to WT. Combinations of two, three, or all four mutations resulted in further attenuation compared to WT EEEV and the individual mutants (Table 2). Of the double mutants, only the C65-69 E71-77 (79.2% survival) or E71-77 3’U11337 (65.2% survival) mutants caused mice to succumb to infection after the primary infection. Mean time to death (MTD) also increased for the mice succumbing to infection for 5’U4&6 (8.2 ± 1.3 days), C65-69 E71-77 (6.9 ± 0.6 days), and E71-77 3’U11337 (6.4 ± 1.5 days) LAVs compared to WT (5.0 ± 1.1 days). Subcutaneous infection with all other LAV candidates yielded 100% survival. Furthermore, mice that survived infection with these viruses did not lose weight, suggesting a high degree of attenuation (Fig 2).

**Table 2:** Mortality rates and mean time to death (MTD) of the LAV candidates in outbred CD-1 mice after primary subcutaneous or intracerebral infection.

Virus	Subcutaneous (sc) Infection			Intracerebral (ic) Infection		
	# surviving mice	Survival (%)	MTD^a^ ± STD^b^ (days)	# surviving mice	Survival (%)	MTD ± STD (days)
WT EEEV FL93-939	2/24^c^	8.3	5.0 ± 1.1	0/8^d^	0	2.4 ± 0.4
5’U4&6	11/24	45.8	8.2 ± 1.3	1/6	16.7	4.2 ± 1.8
C65-69	22/24	91.7	7.5 ± 0.7	2/6	33.3	5.1 ± 0.3
E71-77	1/24	4.2	5.5 ± 0.5	0/8	0	4.3 ± 0.8
3’U11337	21/23	91.3	6.3 ± 0.4	0/8	0	2.9 ± 0.8
5’U4&6 C65-69	24/24	100	N/A	6/6	100	N/A
5’U4&6 E71-77	24/24	100	N/A	5/6	83.3	5 ± 0.0
5’U4&6 3’U11337	24/24	100	N/A	2/6	33.3	5.8 ± 0.9
C65-69 E71-77	19/24	79.2	6.9 ± 0.6	3/6	50	6 ± 0.7
C65-69 3’U11337	23//23	100	N/A	0/6	0	4.9 ± 1.0
E71-77 3’U11337	15/23	65.2	6.4 ± 1.5	0/8	0	3.9 ± 0.5
5’U4&6 C65-69 E71-77	29/29	100	N/A	6/6	100	N/A
5’U4&6 C65-69 3’U11337	30/30	100	N/A	6/6	100	N/A
5’U4&6 E71-77 3’U11337	32/32	100	N/A	6/6	100	N/A
5’U4&6 C65-69 E71-77 3’U11337	33/33	100	N/A	6/6	100	N/A

^a^MTD: Mean time to death of mice succumbing to infection

^b^STD: Standard deviation

^c^Subcutaneous infection: 6–8 independent experiments.

^d^Intracerebral infection: 2–3 independent experiments

In contrast with a sc infection, the YFV-17D LAV is lethal after an ic infection of immunocompetent mice [39], while the VEEV TC-83 LAV is lethal after an ic infection in some mouse strains but not others [40]. Potentially reflecting this retained virulence, both the YFV-17D and the VEEV TC83 vaccines can cause adverse events in humans [41,42]. Therefore, we determined whether the LAV candidates were similarly attenuated after an ic infection. Outbred CD-1 mice were infected ic with equal genomes of each LAV and monitored for morbidity and mortality. WT, E71-77, and 3’U11337 LAV candidates were 100% lethal after ic infection (Table 2). Similar to sc infection, both 5’U4&6 (16.7% survival) and C65-69 (33.3% survival) were attenuated compared to WT. For the double mutant viruses, C65-69 3’U11337 and E71-77 3’U11337 were both 100% lethal within 6 days of infection. By contrast, all mice infected with double mutant viruses containing the 5’U4&6 mutation exhibited increased survival after ic inoculation. 5’U4&6 3’U11337 was the least attenuated (33.3% survival) followed by 5’U4&6 E71-77 (83.3% survival). Interestingly, the combination of 5’U4&6 and C65-69 caused no mortality (Table 2) nor weight loss (Fig 2). Finally, all combinations of three or four mutations resulted in 100% survival (Table 2). These results demonstrate that combining at least three mutations in these virulence loci fully attenuates the EEEV LAV candidates after both sc and ic infection.

### LAV replication in mice

One hypothesis for the ability of the YFV 17D vaccine to induce long-term neutralizing antibody levels suggests that limited vaccine replication early after infection results in strong stimulation of both the innate and adaptive immune responses with few pathological manifestations [43,44]. We hypothesized that with our EEEV LAVs, low levels of virus replication would be required to produce an avirulent virus with potent neutralizing antibody responses. However, combining multiple attenuating mutations into a single LAV could potentially reduce virus replication to the point where effective antibody responses are not generated. To compare *in vivo* replication with the EEEV LAV candidates, we measured replication in both the popliteal lymph node (PLN), an initial site of virus replication, and the serum, 24 hours post infection (hpi).

**Fig 3:**
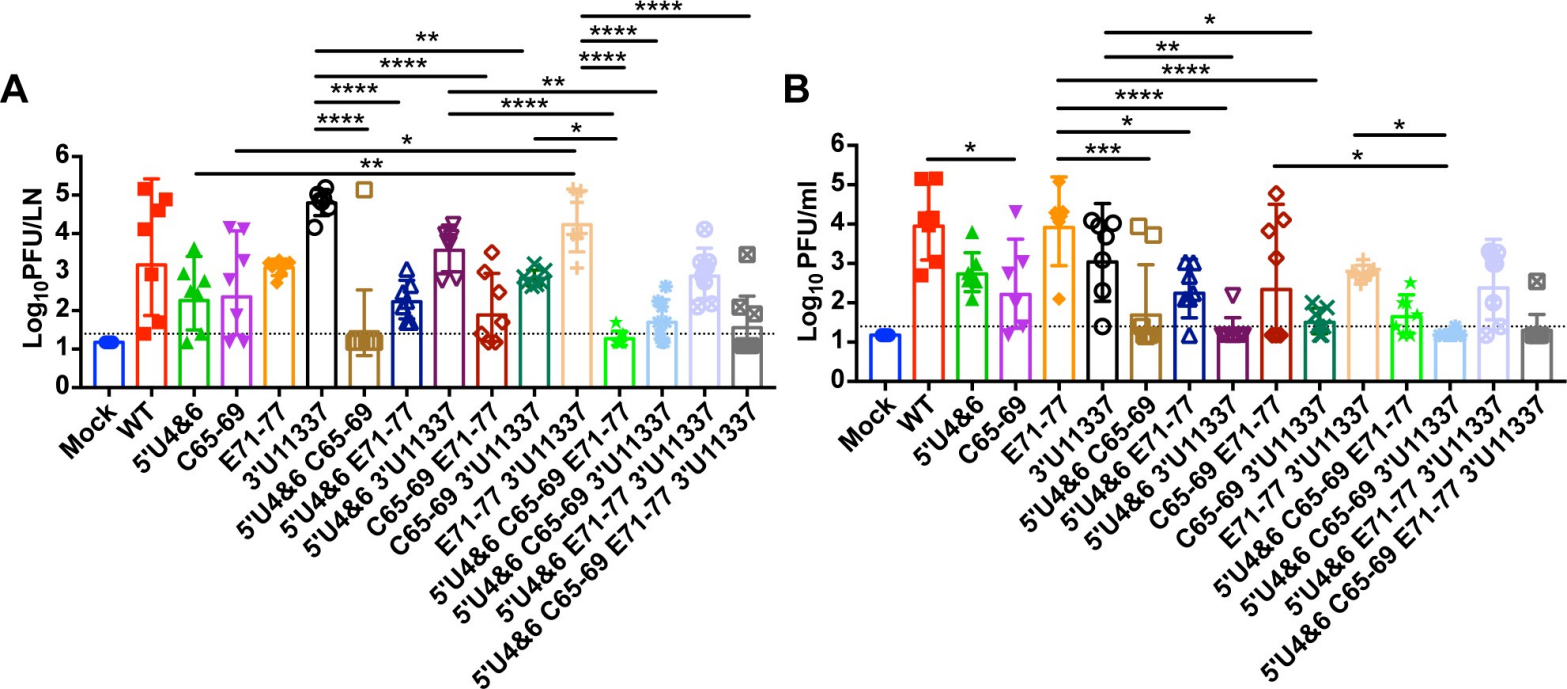
LAV replication in popliteal lymph nodes (PLN) and serum. CD-1 mice were infected with equal genomes of the LAV candidates corresponding to 10^3^ pfu of WT EEEV (WT). At 24hpi, single PLN (A) or serum (B) were harvested and a plaque assay was performed. Each point represents an individual mouse. n = 6–8 mice, 2 independent experiments. Data is log_10_ transformed and the geometric mean (bar) with geometric mean standard deviation graphed and compared between groups. Single mutant LAVs were compared to WT, double mutant LAVs were compared to single mutant LAVs, triple mutant LAVs were compared to the double mutant LAVs, and the quadruple mutant LAV was compared to the triple mutant LAVs. Only comparisons that were significant are represented, all other comparisons were non-significant. *p<0.05, **p<0.01, ***p<0.001, ****p<0.0001 by one-way analysis of variance with Tukey’s multiple-comparison test of log-transformed data.

In the PLN, in the absence of robust myeloid cell replication (viruses with WT sequences in the 3’ UTR), on average ~10^3^ pfu/LN were detected at 24 hpi infection (Fig 3A). The level of virus replication was not uniform between all of the mice suggesting individual variation in the level of miR-142-3p expression between mice or the presence of escape mutants that have eliminated the miR-142-3p binding sites (DW Trobaugh and WB Klimstra manuscript in preparation). Between ~10^2^ and ~10^3^ pfu/LN were detected on average in the PLN after infection with either the 5’U4&6, C65-69, and E71-77 single mutation viruses. 3’U11337 replicated to the highest level (~10^5^ pfu/LN), most likely due to the ability of viruses containing this mutation to replicate in myeloid cells within the PLN [35].

Adding a second mutation to each LAV candidate resulted in a reduction for all of the LAV candidates in mean pfu/LN compared to the single mutation LAV candidates (Fig 3A). For example, combining 5’U4&6 and C65-69 reduced virus replication in the PLN compared to both 5’U4&6 and C65-69 single mutants but not significantly. In fact, only 1 mouse had detectable levels of 5’U4&6 C65-69 in the PLN after 24 hours. Also, adding either 5’U4&6 or C65-69 to the E71-77 LAV backbone reduced virus replication at 24 hpi by 10-fold compared to E71-77 alone, which was not significantly different than either 5’U4&6 or C65-69 alone. A similar reduction was seen after the addition of a second mutation to viruses bearing the 3’U11337 mutation. The 5’U4&6 (P = 0.31) and C65-69 (P<0.01) mutations decreased virus replication by at least 10-fold compared to 11337. There was a slight but not significant reduction in E71-77 3’U11337 virus replication compared to 3’U11337 alone [33,35]. The addition of a third mutation continued to reduce virus replication in the PLN. The 5’U4&6 C65-69 E71-77 virus exhibited the lowest level of virus in the PLN with detectable levels in only 2 of 7 mice. Combining the 5’U4&6 mutation with C65-69 3’U11337 led to a further 10-fold decrease in virus replication compared to C65-69 3’U11337 alone. Similarly, addition of 5’U4&6 to E71-77 3’U11337 reduced virus replication by 10-fold compared to E71-77 3’U11337 alone. Finally, only 3 of 8 mice had detectable levels of the virus with four mutations in the PLN.

We also measured virus levels in the serum at 24 hpi to determine whether the mutants produced similar levels of serum viremia compared to WT. At 24 hpi, WT and E71-77 had the highest level of serum viremia (~10^4^ pfu/ml) compared to all of the other mutants (Fig 3B). Serum viremia of the other single mutants was reduced either 100-fold (C65-69 (P<0.05)) or 10-fold (5’U4&6 and 3’U11337) compared to WT. Serum viremia of the LAVs containing two mutations were further reduced by 10- to 100-fold from WT and E71-77. 5’U4&6 3’U11337 had the lowest level of serum viremia, which was detected in only a single mouse. The highest level of serum viremia for the double mutants was in the C65-69 E71-77 group. Similar to virus replication in the PLN, serum viremia was further reduced with the addition of the third and fourth mutations. Only a single mouse infected with 5’U4&6 C65-69 3’U11337 or 5’U4&6 C65-69 E71-77 3’U11337 had serum viremia in contrast to mice infected with 5’U4&6 E71-77 3’U11337 where only a single mouse was below the limit of detection. Together, these results demonstrate that increasing the number of mutations in the vaccine candidates led, generally, to reduction in early virus replication in the PLN and serum viremia compared to WT EEEV. However, considerable variability was encountered likely due to the specific effect of mutations on myeloid cell infection, virus spread, and IFN resistance.

### Myeloid cell replication in PLN is required for systemic IFN production

We have previously demonstrated that the 3’U11337 single mutation virus induced high levels of serum IFN-α/β compared to WT EEEV within 12 hpi infection, and this serum IFN-α/β was required for the attenuation of 3’U11337 *in vivo* [35]. Furthermore, combination of the E71-77 and 3’U11337 mutations resulted in even greater induction of serum IFN-α/β at 12 hpi, possibly by increasing the access of myeloid cell replication-competent viruses to the PLN and spleen [33,35]. Production of IFN-α/β early after virus infection is an important factor in B and T cell activation and differentiation leading to robust acquired immune responses [45,46]. Therefore, we next determined whether the LAV candidates would induce serum IFN-α/β independently of the 3’U11337 and E71-77 mutations or whether these mutations were required for IFN-α/β production.

**Fig 4:**
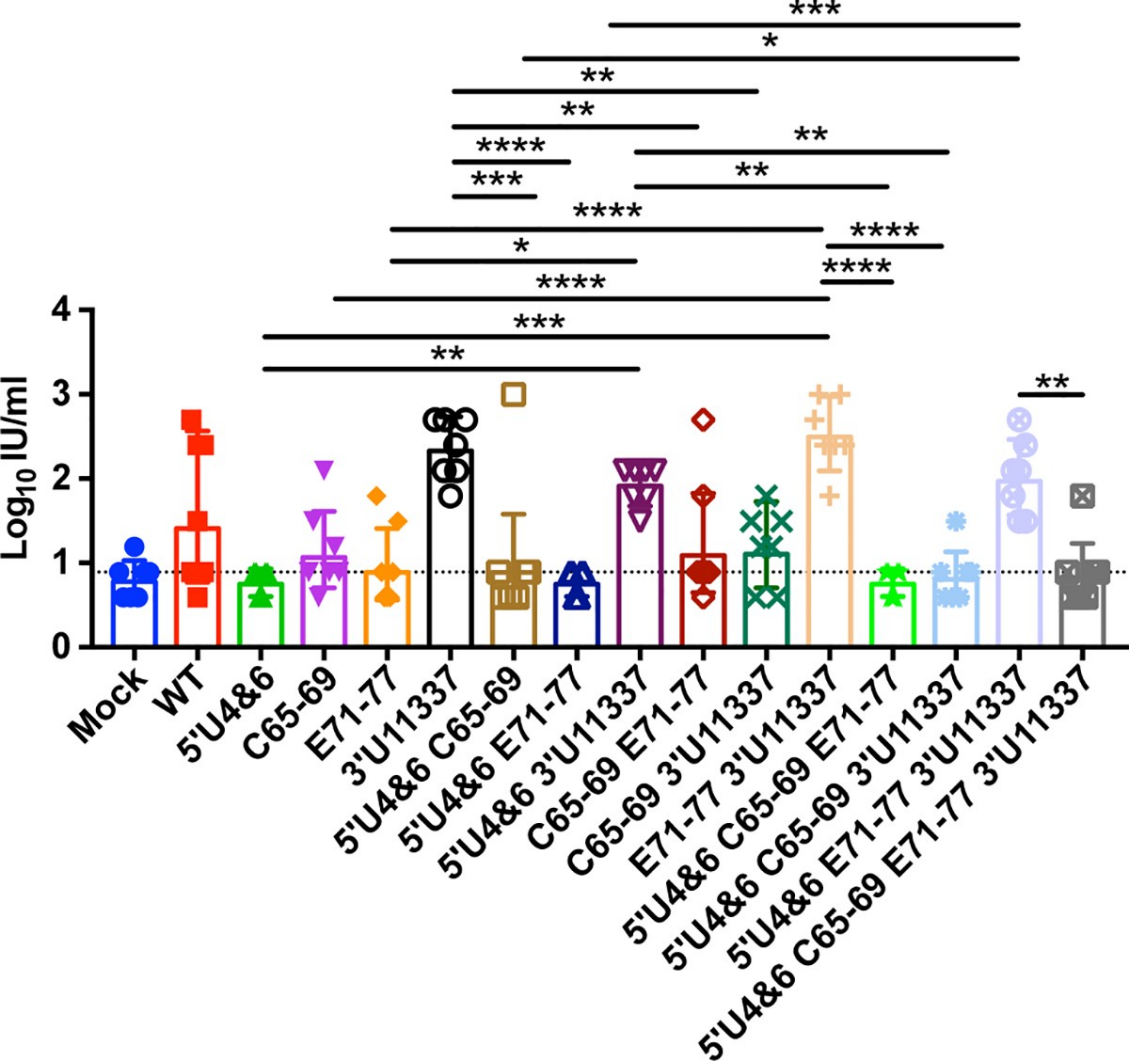
Systemic IFN production after LAV infection. CD-1 mice were infected with equal genomes of the LAV candidates corresponding to 10^3^ pfu of WT EEEV. At 24hpi, serum was collected for a biological IFN assay. Each point represents an individual mouse. n = 6–8 mice, 2 independent experiments. Data is log_10_ transformed and the geometric mean (bar) with geometric mean standard deviation graphed and compared between groups. Single mutant LAVs were compared to WT, double mutant LAVs were compared to single mutant LAVs, triple mutant LAVs were compared to the double mutant LAVs, and the quadruple mutant LAV was compared to the triple mutant LAVs. Only comparisons that were significant are represented, all other comparisons were non-significant. Dotted line indicates limit of detection for assay. *p<0.05, **p<0.01, ***p<0.001, ****p<0.0001 by one-way analysis of variance with Turkey’s multiple-comparison test of log-transformed data.

Serum was harvested from mice at 24 hpi and serum IFN-α/β was quantitated by bioassay. As expected, 3’U11337 elicited the highest and most consistent levels of serum IFN-α/β of the single mutant viruses (Fig 4) [35]. In contrast to our previous data [35,47], some WT infected mice had serum IFN-α/β levels at 24 hpi suggesting variability in the outbred CD-1 population or the generation of escape mutants that can replicate in myeloid cells (DW Trobaugh and WB Klimstra manuscript in preparation). When double mutants were considered, the 3’U11337 mutation was required for consistent IFN-α/β production between mice in a group and levels were slightly but not significantly augmented by the presence of E71-77. IFN-α/β was suppressed compared to E71-77 3’U11337 when E71-77 was combined with any mutation other than 3’U11337. With three or four mutation viruses, 3’U11337 was required for detection of IFN-α/β in any animals and the 5’U4&6 E71-77 3’U11337 virus elicited the highest and most consistent levels. In general, higher levels of PLN replication (Fig 3A) were reflected in higher serum IFN-α/β levels (Fig 4) between viruses suggesting that PLN replication is a major factor in induction of the IFN-α/β response. Overall, PLN replication, which 3’U11337 promotes most directly, is required for consistent serum IFN-α/β production by the LAV candidates and the E71-77 mutation may sustain these levels in the presence of another attenuating mutation.

### LAV-immunized mice are protected from sc and aerosol EEEV challenge

**Fig 5:**
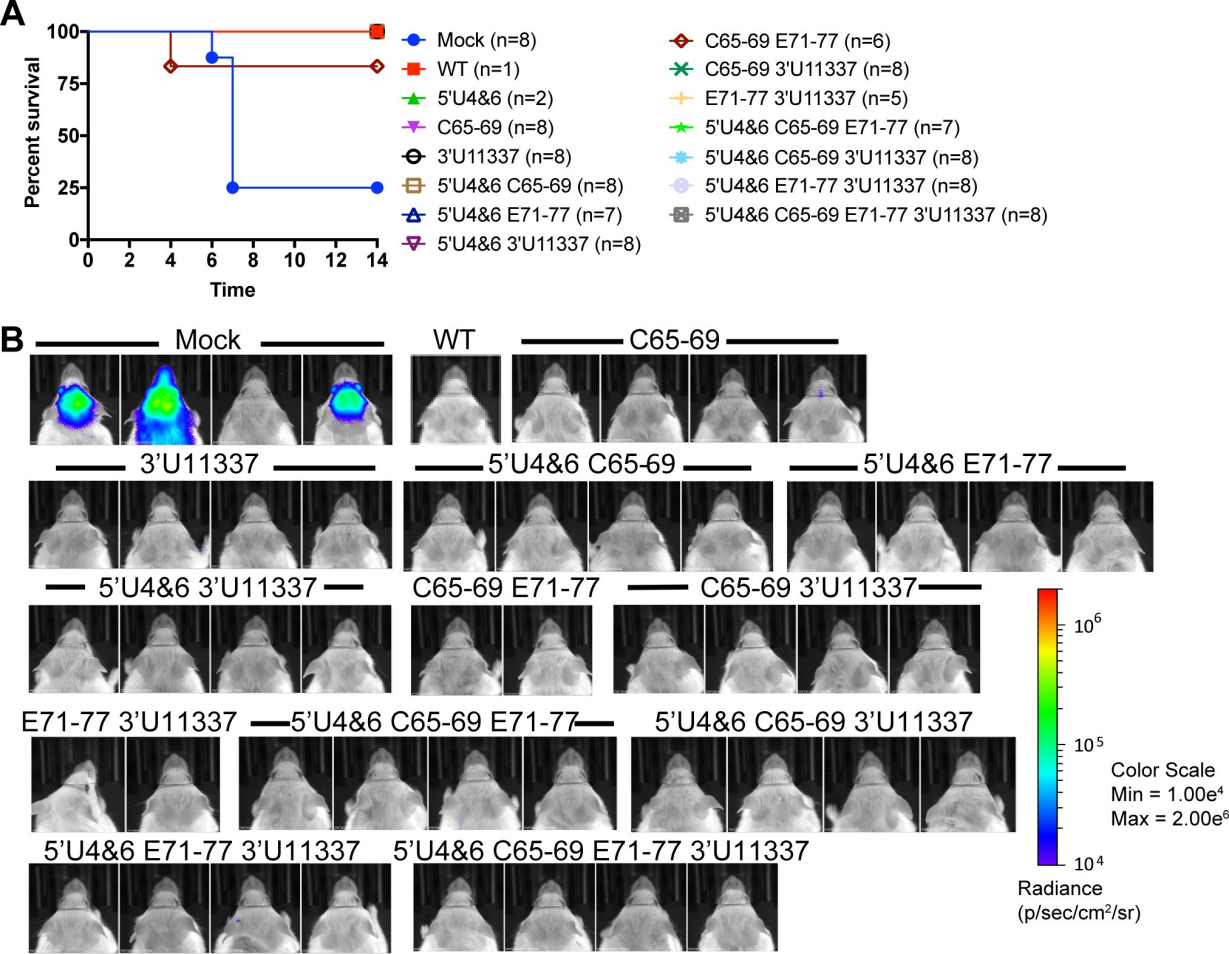
LAVs protect against high dose subcutaneous EEEV challenge. A) On day 22 post primary LAV infection, CD-1 mice that survived were challenged subcutaneously with 104–10^5^ pfu of EEEV-nLuc TaV virus. n = number of total mice challenged from 2 independent experiments. B) IVIS images on day 6 of mice challenged with 10^4^ pfu EEEV-nLuc TaV from a single experiment. All images are set to the same scale.

Three weeks after the primary sc vaccination, mice that survived were challenged either sc (10^4^ or 10^5^ pfu) or with a standard dose (50–100 LD_50_) aerosol infection of WT EEEV FL93-939 encoding nanoLuciferase (nLuc) as a self-cleavable protein, which is similarly virulent to the unmodified parental FL93-939 strain [48]. Mice were monitored daily for morbidity and mortality, and on day 4 (aerosol) or day 6 (sc) post challenge, mice were imaged using an IVIS Spectrum-CT *in vivo* imager to quantify virus replication in the brain. After sc challenge, mock-infected controls that succumbed to the challenge (6 of 8 mice) died by day 7 (Fig 5A) and had high levels of virus replication in the brain detected by IVIS imaging (Fig 5B). The single C65-69 E71-77 immunized mouse that died after sc challenge on day 4 after infection did not completely recover prior to challenge. All other LAV-vaccinated mice survived the high dose sc challenge with no observable weight loss after infection (S1 Fig) demonstrating that the LAVs induce complete protection against a sc challenge.

**Fig 6:**
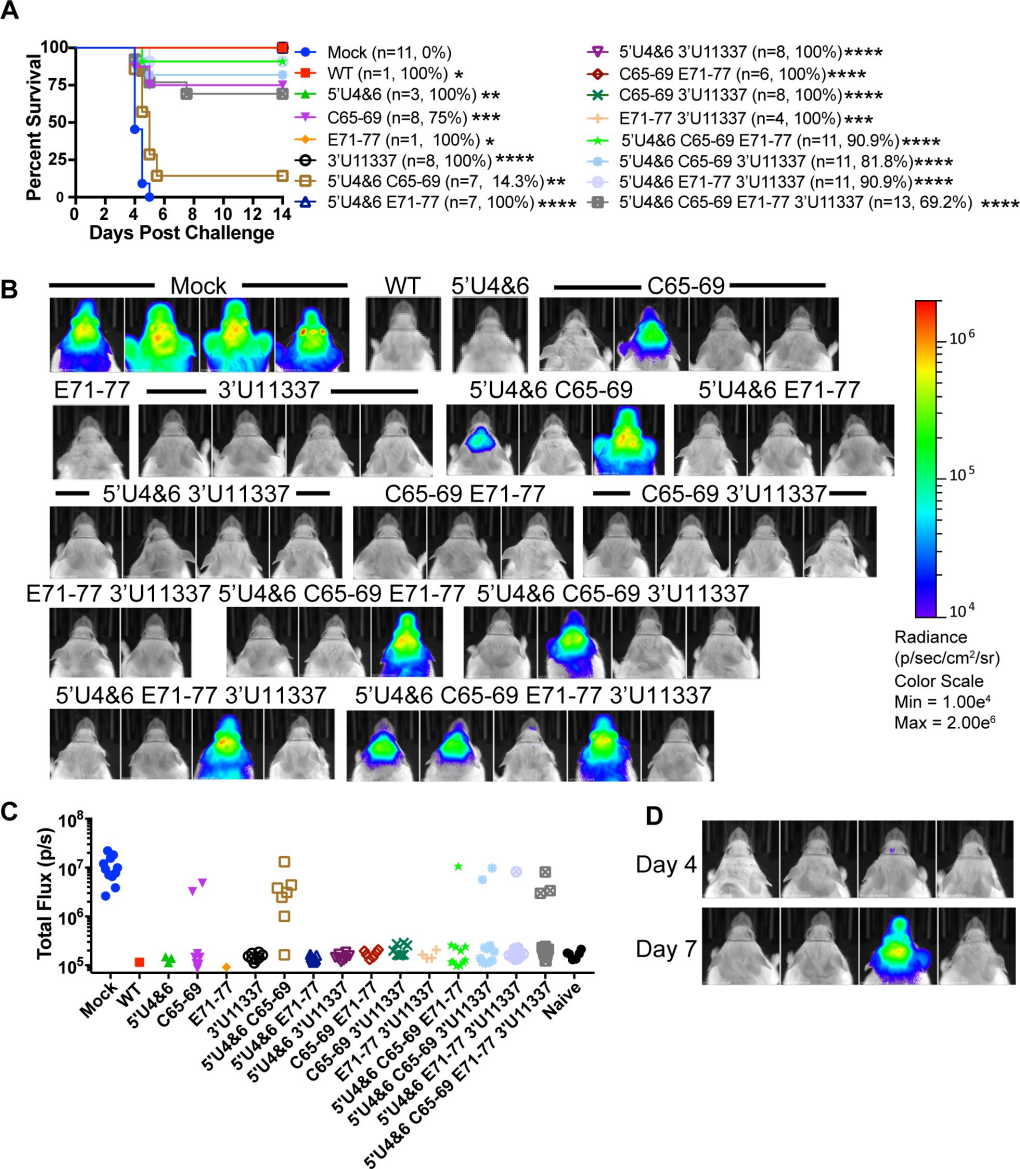
LAVs protect against an aerosol challenge. A) CD-1 mice that survived the primary LAV infection were challenged on D22 post primary infection. In parentheses are the numbers of mice surviving each LAV that were challenged by aerosol and the percent survival for each group. Mock mice were mock vaccinated controls for the experiment. 3 independent aerosols were performed using 100LD_50_ EEEV expressing nLuc. Calculated doses for each aerosol were 73,554 pfu,96,118 pfu, and 78,814 pfu. Only the triple mutant and quadruple mutant LAVs received the 78,814 pfu aerosol dose. *p<0.05, **p<0.01, ***p<0.001, ****p<0.0001, Log-Rank Test compared to mock. B) Representative IVIS images on day 4 of one aerosol challenge experiment. All IVIS images are set to the scale shown on right. C) Luciferase quantification of day 4 IVIS images represented as total flux (p/s). Naive mice were not challenged during the experiment and used for background total flux levels. D) IVIS images from 5’U4&6 C65-69 E71-77 3’U11337 LAV group on day 4 (top row) and day 7 (bottom row). All images in figure are set to the same scale (B).

All aerosol-challenged control mice succumbed to infection within 5 days (Fig 6A). On day 4, mock-immune mice had 100-fold more virus replication as measured by IVIS quantitation of nLuc activity in the brain compared to non-challenged control mice (Fig 6B and 6C). In general, mice that survived the aerosol challenge did not have discernable weight loss (S2 Fig) or detectable levels of virus replication by IVIS in the brain on day 4 post challenge (Fig 6B and 6C). Surprisingly, the 5’U4&6 C65-69 vaccinated mice rapidly succumbed to aerosol challenge with only 1 out of 7 mice surviving (14.3%) with all of the sick mice having 10-100-fold increases in virus replication in the brain *versus* uninfected controls. In contrast, six out of eight mice (75% survival) vaccinated with the single mutant C65-69 survived the aerosol challenge; the two sick mice had 100-fold increases in virus replication. Only 1 mouse vaccinated with the 5’U4&6 C65-69 E71-77 or 5’U4&6 E71-77 3’U11337 mutants succumbed to the aerosol challenge. Interestingly, there was a delay in virus replication in the brain of a 5’U4&6 C65-69 E71-77 3’U11337 vaccinated mouse that succumbed to infection. The single mouse had low-undetectable levels of nLuc in the brain on day 4 post infection, but by day 7, virus replication was 100-fold higher (Fig 6D). Together, the challenge results demonstrate that the EEEV LAV candidates protect uniformly against a sc challenge and the three-four mutation viruses protect partially to completely against the aerosol challenge.

### Association of neutralizing antibody levels with protection against an aerosol challenge

**Fig 7:**
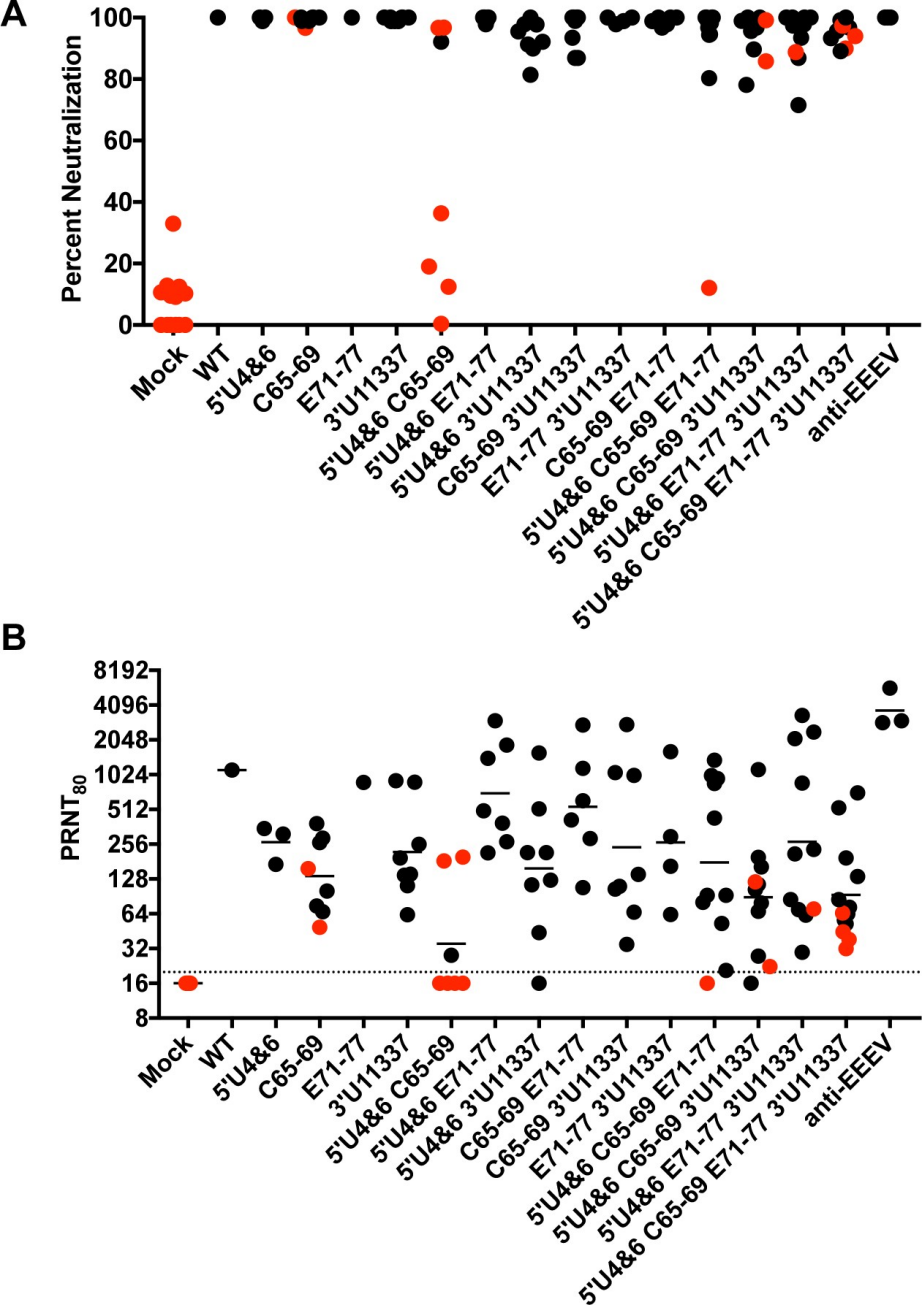
LAVs induce high levels of neutralizing antibodies that protect against aerosol challenge. Serum was collected on D21 post primary LAV infection and screened for neutralization antibody levels using a SINV-EEEV chimeric virus encoding the EEEV FL93 structural proteins. (A) Percent neutralization at 1:20 dilution of serum. (B) Reciprocal PRNT_80_ values for each mouse are graphed. Each black circle indicates a single mouse that survived aerosol challenge. Each red circle indicates a mouse that died after aerosol challenge. The black line represents geometric mean PRNT_80_ value and the dotted line indicates limit of detection of the assay.

Little is known regarding the correlates of protection required for, not only protection from an EEEV infection, but from all alphaviruses [24,49]. The inactivated EEEV vaccine given to at-risk workers uses a PRNT_80_ neutralizing antibody value of 1:40 as demonstrative of adequate protection; however, this has not been experimentally validated [24]. Since some vaccinated mice succumbed to the aerosol challenge, we determined whether or not we could identify a serum antibody neutralization value required for protection from an aerosol infection. Serum was harvested from vaccinated mice at 3 weeks post inoculation, one day prior to aerosol challenge. Sera were tested against a chimeric virus encoding the Sindbis nonstructural genes and the structural genes of WT EEEV FL93-939 [50], derived from the challenge virus, for neutralizing activity in a standard PRNT assay using commercially available anti-EEEV sera as a control. At a 1:20 dilution of serum, mice immunized with the viruses containing only a single mutation all exhibited close to or achieved 100% neutralization (Fig 7A). Not all mice that were vaccinated with viruses containing more than one mutation had complete neutralization at a 1:20 dilution. In fact, substantial variability was seen in the mice vaccinated with viruses with two, three or four mutations and some sera from these mice exhibited less than 80% neutralization. This suggests that attenuation of these viruses may affect the production of neutralizing antibodies. Also, the presence of neutralizing activity at a 1:20 dilution did not guarantee protection from aerosol challenge. Notably, mice immunized with C65-69, 5’U4&6 C65-69, 5’U4&6 C65-69 3’U11337, and 5’U4&6 C65-69 E71-77 3’U11337 LAVs exhibited neutralizing activity at a 1:20 dilution but still succumbed to the aerosol infection and several animals with 100% neutralization also succumbed. Interestingly, all but one animal that succumbed included the C65-59 mutation that decreases shutoff of cellular transcription.

Variability was also seen in PRNT_80_ values from the vaccinated mice. The highest average PRNT_80_ values were elicited by 5’U4&6 E71-77 and C65-69 E71-77 double mutation viruses. Average PRNT_80_ values for the three or four mutation viruses were generally equivalent to or lower than the single and double mutation viruses (with the notable exception of 5’U4&6 C65-69). The majority of immunized mice that had PRNT_80_ values below the LOD did not survive the standard dose aerosol challenge (Fig 7B), however, two did survive. All mice with a PRNT_80_ above 1:256 survived standard dose aerosol challenge regardless of the virus used for vaccination. Below this PRNT_80_ value, there was not a direct association of neutralization capacity with protection. For example, with C65-69 and 5’U4&6 C65-69, some mice that had a PRNT_80_ value above 1:128 also succumbed to disease. Protective responses in mice with low serum PRNT_80_ values suggest that other immune responses such as production of mucosal IgA or CD4^+^/CD8^+^ T cell activation may also play a role in protection from EEEV aerosol challenge.

### LAV-immunized mice are protected against a high dose aerosol challenge

**Fig 8:**
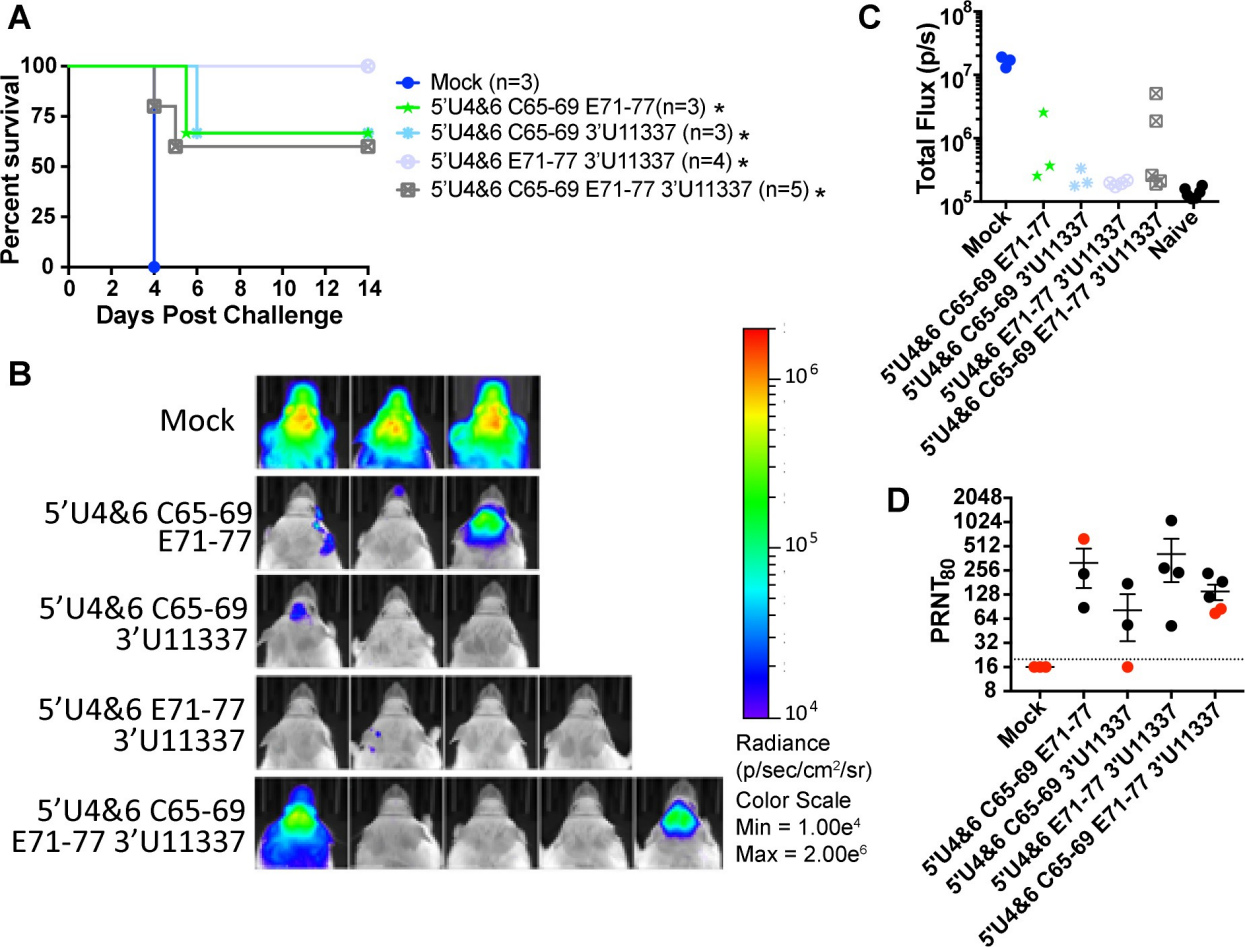
LAVs protect against a high dose EEEV aerosol challenge. Three to five CD-1 mice were challenged at D22 post primary infection with a > 1000 LD_50_ dose of EEEV expressing nLuc (calculated dose: 512,066 pfu) A) Survival of mice following aerosol challenge. Number of mice challenged is indicated in parentheses. * P<0.5, Log-Rank Test compared to mock unvaccinated mice B) IVIS images of LAV mice collected on day 4 post challenge. C) Quantification of IVIS images represented as total flux (p/s). Naive mice were not challenged during the experiment and used for background total flux levels. D) Serum collected on D21 prior to aerosol challenge were screened for neutralizing antibodies. Reciprocal PRNT_80_ values are graphed. Each dot represents a single mouse and red dots indicate mice that succumbed to the aerosol challenge. The dotted line indicates limit of detection.

Finally, to assess the dose-responsiveness of aerosol protection afforded by the viruses with the most desirable attenuation properties, we immunized mice as above with the 3 or 4 mutation viruses and subjected them to a high dose aerosol challenge (>1000 LD_50;_ Fig 8A). All vaccine viruses provided over 50% protection from mortality (P<0.05 versus mock) and weight loss (S3 Fig) and the 5’U4&6 E71-77 3’U11337 protected all of the mice. Each LAV vaccinated mouse that succumbed to the high dose aerosol infection had 10-fold lower levels of nLuc signal in the brain compared to mock mice on day 4 (Fig 8B and 8C) suggesting there is some low-level protection afforded by these LAVs but not complete protection. In this case, a mouse succumbed to challenge with a neutralization titer of >1:512 (Fig 8D) suggesting greater stringency than the 50-100LD_50_ challenges. Together, our results demonstrate that the LAVs containing three or four mutations are attenuating *in vivo* via both sc and ic infection, protect against sc infection, and generate sufficient immune responses for protection from stringent aerosol challenge in vaccinated mice.

### LAVs replication in myeloid cells is required for an inflammatory response and T cell responses

**Fig 9:**
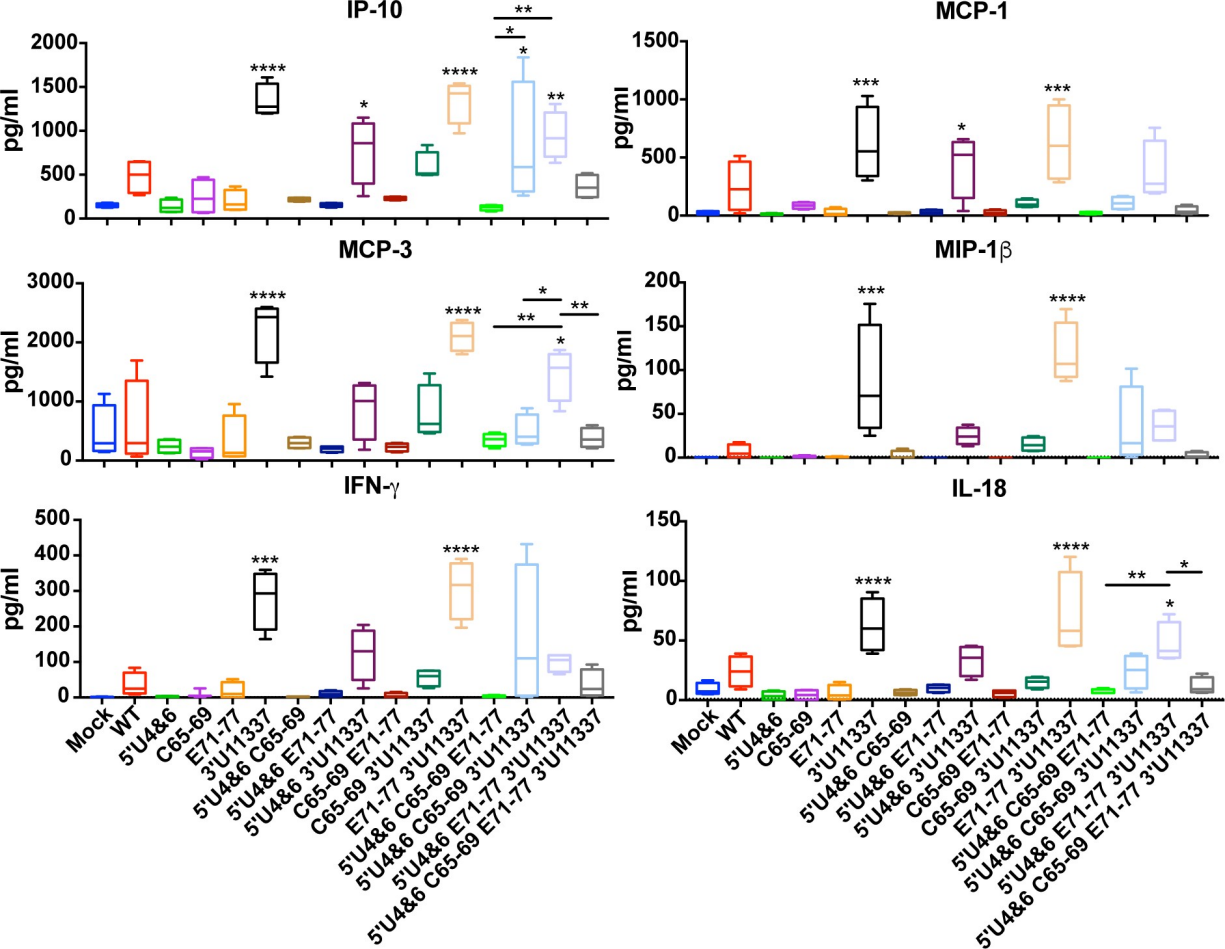
LAVs competent for myeloid cell replication induce greater systemic cytokine responses. CD-1 mice were infected with equal genomes of the indicated LAV and at 24 hours post infection, serum was collected for Luminex analysis. Asterisks above each box and whisker plots indicate significant difference compared to mock. Bars indicate significant differences between triple and quadruple mutant LAVs only. *P<0.05, **P<0.01, ***P<0.001, ****P<0.0001, by one-way analysis of variance with Turkey’s multiple-comparison test. N = 4 mice.

To further differentiate between the 3 and 4 mutant LAV candidates to identify and optimal vaccine candidate, we investigated whether these viruses induced different host immune responses. Since we have already determined that myeloid cell replication is required for serum IFN production (Fig 4), we wanted to determine whether myeloid cell replication was required for the production of inflammatory cytokines and chemokines during immunization (Fig 9). LAVs that were competent for myeloid cell replication (11337 mutation) elicited higher levels of IP-10, MCP-1, MCP-3, MIP-1β, IFN-γ, and IL-18 when compared to mock mice and the LAVs without the mutation. When comparing the 3 and 4 mutant LAVs, 5’U4&6 E71-77 3’U11337 immunization resulted in significantly higher levels of IP-10 and MCP-3 compared to the other 3 or 4 mutant LAVs. 5’U4&6 C65-69 E71-77 had the lowest cytokine response of all the 3 and 4 mutant LAVs most likely due to its inability to replicate in myeloid cells. Furthermore, the addition of C65-69 to 5’U4&6 E71-77 3’U11337 resulted in lower cytokine responses compared to 5’U4&6 E71-77 3’U11337. All other cytokine and chemokine responses were not significantly different from background.

**Fig 10:**
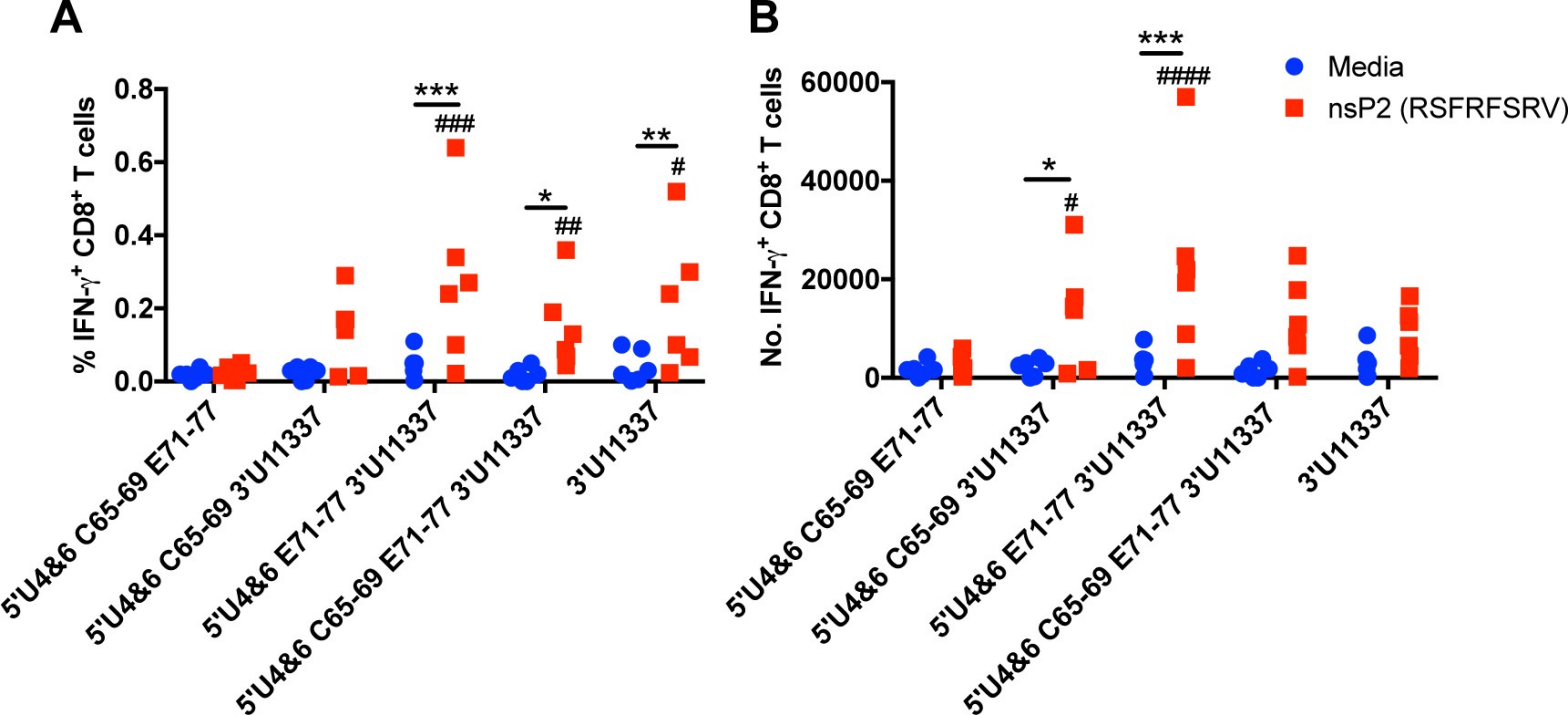
LAVs competent for myeloid cell replication induce early epitope-specific T cell responses in spleen. C57BL6 mice were immunized with equal genomes of the LAVs in both rear footpads. On day 6, splenocytes were stimulated with an EEEV-specific nsP2 peptide (1μM) for 5 hr. A) Percent of IFN-γ^+^ CD8^+^ T cells in spleen. B) Number of IFN-γ^+^ CD8^+^ T cells in spleen. *p<0.5, **p<0.01, ***p<0.001 by two-way analysis of variance with multiple comparisons using the Bonferroni method comparing between media and nsP2 stimulation of each LAV immunization. #p<0.05, ##p<0.01, ###p<0.001, ####p<0.0001 by two-way analysis of variance with multiple comparisons using the Bonferroni method comparing nsP2 peptide stimulation of each LAV with 5’U4&6 C65-69 E71-77n = 6 mice, 2 independent experiments.

Next, we wanted to further evaluate the 3 and 4 mutant LAV candidates to determine whether they induced quantitatively different CD8^+^ T cell responses after immunization. We immunized C57Bl/6 mice with equal genomes of the 3 or 4 mutant LAVs sc in both rear footpads, and early epitope-specific CD8^+^ T cell responses in the spleen were measured using an EEEV-specific peptide (RSFRFSRV) located in the nsP2 protein. On day 6 post immunization, splenocytes were harvested to quantify IFN-γ^+^ CD8^+^ T cell responses. The LAVs that were competent for myeloid replication (11337 mutation) had higher frequencies (Fig 10A) and numbers (Fig 10B) of EEEV-specific IFN-γ^+^ CD8^+^ T cells (nsP2) compared to media control and the only non-myeloid tropic LAV, 5’U4&6 C65-69 E71-77. There were no significant differences in frequency or number of IFN-γ^+^ CD8 T cells when comparing between the LAVs that replicate in myeloid cells (5’U4&6 C65-69 3’U11337, 5’U4&6 E71-77 3’U11337, or 5’U4&6 C65-69 E71-77 3’U11337). However, a trend for greater CD8^+^ T cell abundance was evident with the 5’U4&6 E71-77 3’U11337, which induced significantly higher cytokine levels in the serum and was most protective from aerosol challenge. Together, these data demonstrate that incorporating myeloid cell replication into the LAVs by eliminating the miR-142-3p binding sites (11337) induces a more robust inflammatory response in the serum and T cell response in the spleen.

## Discussion

LAVs are an effective tool in combating medically important pathogens. However, LAVs can induce adverse events in some individuals limiting their use and distribution. Historically, LAVs have been generated by blind passaging in cell culture or animal models until attenuation was achieved. This serial passaging led to the accumulation of mutations in the virus genome that decreased virus virulence. While the mutations could be identified by sequencing, their specific mechanisms of attenuation were rarely known. This has remained true for very widely used LAVs such as the YFV 17D LAV [9] or the poliovirus LAV [14], which have been given to hundreds of millions of individuals. For example, with the exception of a substitution to positive charge in the DIII loop of the YFV E protein that confers enhanced interactions with heparan sulfate [51], the molecular attenuation mechanisms conferred by some or all of the 31 specific mutations in YFV 17D LAV have not been well characterized [9]. In addition, attenuating mutations selected by blind passage of LAVs are not designed to resist reversion and often involve single nucleotide changes [3,10–12]. This can lead to rapid reversion to non-attenuated phenotypes [15,40].

Here, we have studied the effects upon attenuation, lymphoid tissue tropism, elicitation of neutralizing responses and protection from sc or aerosol WT EEEV challenge of four mutant loci in the genome of EEEV whose mechanisms of action are known and whose mutant sequences are specifically designed to resist reversion. These loci/mutations were chosen to increase IFN sensitivity (5’U4&6) [28]; decrease HS binding and neurovirulence and increase virus particle access to lymphoid tissue (E71-77) [33]; decrease shutoff of host cell transcription, thereby, increasing host cell responses to infection and potentially antigen presentation in infected cells (C65-59) [32]; and eliminate miR-142-3p restriction, thus increasing myeloid cell replication, direct antigen presentation and cytokine responses (3’U11337) [35].

Combinatorial mutation of these known virulence loci had no effect on virus replication in the Vero mesenchymal cell line (Fig 1) demonstrating that these mutations have no direct inhibiting effect upon virus genome replication in mammalian cells. The mutation that disrupts HS binding, E71-77, limits growth for all of the viruses containing this mutation, due to inefficient cell binding and lower infectivity in vitro [33,34]. Growth kinetics of the viruses should be different in myeloid cells due to the presence or absence of the miR-142-3p binding sites [35]. In addition, Vero cells are defective in IFN-α/β production and, thus, the effects of IFN-mediated antiviral activity are not accounted for in our *in vitro* testing.

In contrast with Vero cells, in mice, incorporation of at least three mutations in virulence loci rendered the viruses completely attenuated (no mortality or morbidity) after either a sc or ic infection. Avirulence from an ic inoculation route suggests a degree of safety beyond that provided by passage-derived live attenuated vaccines such as YFV 17D or TC83 both of which can cause morbidity or mortality in mice from this route [39,40]. However, additional experiments will be required to determine if murine avirulence is strongly associated with avirulence in primates and whether or not LAV candidates highly attenuated in mice could be given to immunocompromised or juvenile human populations, which are contraindicated even for safe LAVs such as YFV 17D [16].

We have also demonstrated that all but one mouse that survived the vaccination process were completely protected (no morbidity or mortality) from sc challenge with EEEV. Even the 5’U4&6 C65-69 double mutant virus, which elicited very low neutralization titers (most mice <80% at 1:20 dilution of serum), was completely protective. Therefore, as with current use of the inactivated EEEV IND vaccine to protect at-risk humans, detectable neutralization titer is a reasonable measure of protection from the natural route of infection in mice. Standard and high dose aerosol challenge yielded substantially less protection, and this was distributed through all mutation groups (1–4 mutations); although, of the 6 double mutation viruses, 5 provided complete protection from standard dose aerosol challenge. Among 3 or 4 mutation viruses the 5’U4&6 E71-77 3’U11337 virus elicited the highest level of protection to >100LD_50_ challenge (1 of 11 mice succumbed) and the virus was completely protective even after even a >1000LD_50_ aerosol challenge. This minor variability in protective responses after aerosol challenge may reflect immune response differences between individual mice in the outbred CD-1 model used here.

A PRNT_80_ value >1:512 was associated with complete protection against all aerosol doses while the relationship of neutralization titer to protection from aerosol challenge at lower PRNT_80_ levels was not as clear. However, all mice that exhibited less than 70% plaque neutralization at a 1:20 dilution of serum did succumb to aerosol challenge. A direct association of replication competence *in vivo* with protection is implied by the fact that viruses with two mutations produced higher neutralization titers and greater protection against challenge than viruses with three or four mutations and their replication in the PLN and serum was generally higher. However, reactogenicity profiles with the two mutation viruses are possibly unacceptable in that mice succumbed to the vaccination dose with the E71-77 3’U11337 and C65-59 E71-77 viruses and none were avirulent after ic inoculation. Similarly, the 5’U4&6 C65-69 E71-77 3’U11337 four mutation virus showed less protection after aerosol challenge than any of the three mutation viruses. Future work will examine whether these LAVs induced different mucosal immune responses that can provide sterilizing immunity upon an aerosol infection.

The predicted activity of several of the mutations was recapitulated *in vivo*. The 3’U11337 mutation that greatly increases myeloid cell infection by EEEV *in vitro* and *in vivo* [35] did increase PLN replication and IFN-α/β in serum in most combinations. In the context of vaccines, this is likely to increase immune responses in a number of ways including infection of antigen presenting cells, increasing multiple aspects of immune stimulation. Consistent with this, we observed that incorporation of this mutation into the LAV induced higher pro-inflammatory cytokine levels in sera and higher frequency and numbers of virus-specific CD8^+^ T cells at 6 days post-infection compared to the non-myeloid tropic LAVs. However, neutralization titer values were not highly reflective of the presence of this mutation as the highest average PRNT_80_ values were produced by double mutants lacking this mutation, potentially as a consequence of lower IFN-α/β induction by these viruses *in vivo*.

Similarly, we predicted that the 3’U11337 mutation would increase PLN replication and serum IFN-α/β induction in most contexts. The single mutation did increase PLN replication versus the WT virus but this was not associated with higher serum IFN-α/β at 24 hpi. Supporting the prediction, the combination of E71-77 with 3’U11337 exhibited the highest PLN replication and IFN-α/β production of the double mutants and also, this combination was reflected in highest PLN replication and IFN-α/β induction among the triple mutants. Previous models of infection with single mutant viruses assayed IFN titers at 12 hpi. By 24 hpi, escape mutants may be generated during WT EEEV infection that have increased tropism for myeloid cells (DW Trobaugh and WB Klimstra, manuscript in preparation). This myeloid cell replication would then lead to higher serum IFN-α/β production similar to 3’U11337 [35,47].

Notably, presence of the C65-69 mutation appeared to result in incomplete aerosol challenge immunity in several contexts. The mutation deletes a nuclear localization signal, greatly reduces shutoff of host cell transcription after infection [32] and likely interferes with any other nuclear activities of the capsid protein [52,53]. However, it has no effect upon replication *in vitro* in the absence of an IFN-α/β response. It could be predicted that, *in vivo*, this mutation would increase antigen presentation in infected cells and, possibly increase serum IFN responses. In mice, viruses within mutation groups (e.g. 1 mutation versus 2, 3 or 4) that possessed this mutation showed generally lower replication in the PLN and lower serum IFN-α/β induction. This mutation provided the poorest protection and neutralization titers when present alone or in combination with the 5’U4&6 mutation, and this combination of mutations was also the only double mutant exhibiting complete avirulence after ic inoculation. Furthermore, neutralization titers and protection were greatest among 3 mutation viruses when this mutation was omitted. However, it also should be noted that C65-69 was possessed by several two or three mutation viruses that were highly protective. Therefore, it appears that effects of this mutation on protective responses may be more reflective of a context-dependent effect upon replicative fitness *in vivo* rather than the specific activity of the mutant locus.

The 5’U4&6 mutation did not appear to have a distinct or consistent effect upon virus replication, IFN-α/β induction, neutralization titer or protection *in vivo*. For example, it was present in the double mutant with the lowest neutralization titers as well as the triple mutant with the highest. Not surprisingly, the primary impact of the mutation: increased sensitivity to genome binding by the IFN induced antiviral effector protein IFIT-1 [28], does not have a readily apparent association with immunogenicity. However, the mutation clearly provided an attenuating effect in the context of the single mutant or the triple mutants. Therefore, this mutation is possibly not associated with modulation of the immune response beyond its attenuating properties.

Ultimately, our data suggest that informed mutation of virulence loci can generate safe and effective LAVs even for viruses with the extreme virulence of EEEV. Our studies do suggest that knowledge of particular attenuation mechanisms can provide some predictive value regarding attenuation and immunogenicity *in vivo*. However, beyond what may be a specific circumstance with WT EEEV related to its unusual lack of tropism for myeloid cells, informed derivation of a LAV will require empirical assessment of the balance between attenuation and multiple aspects of immunogenicity. Our results also suggest that attenuation and immunogenicity must be considered as separate aspects of informed vaccine design. In our case, with one exception, double mutant viruses elicited the highest and most consistent neutralization responses and provided 100% protection against normal dose aerosol challenge. However, several were unacceptably virulent from a sc inoculation and only one was avirulent from an ic inoculation. Therefore, the margin of safety for these viruses may not be acceptable in humans. Three and four mutation viruses were completely avirulent from both sc and ic inoculation, but none provided complete protection from aerosol challenge and neutralization titers were not as consistent or high on average as with the double mutants. However, only one mouse died in four aerosol challenge experiments after immunization with 5’U4&6 E71-77 3’U11337 and all mice did survive the high dose aerosol challenge. Interestingly, among the three or four mutation viruses, this virus elicited the highest levels of MCP-1, MCP-3 and IL-18 and showed a trend towards production of higher numbers of CD8^+^ T cells, possibly underlying its superior protective efficacy.

Since the extent to which murine results are applicable to humans is not clear, we propose that a range of viruses exhibiting ic and/or sc avirulence as well as a high degree of aerosol protective efficacy be tested in non-human primate models, including the sc-avirulent, aerosol-protective double mutants with highest neutralization titers (5’U4&6 E71-77 and C65-69 E71-77) as well as the 5’U4&6 E71-77 3’U11337 triple mutant and the 5’U4&6 C65-69 E71-77 3’U11337 quadruple mutant. Furthermore, complete characterization of the avirulence/immunity relationships in NHPs will be required including examination of multiple aspects of the disease profile such as febrile responses after immunization and immune response generated by the vaccines such as serum antibody class, subclass, presence of antibodies at respiratory mucosal surfaces, compete cytokine analysis, stimulation of CD4^+^ and CD8^+^ T cells and their effector activities, and assessment of their relationship to protective efficacy.

## Materials and methods

### Ethics statement

All animal procedures were carried out under approval of the Institutional Animal Care and Use Committee of the University of Pittsburgh in protocols 15066059 and 18073259. Animal care and use were performed in accordance with the recommendations in the Guide for the Care and Use of Laboratory Animals of the National Research Council. Approved euthanasia criteria were based on weight loss and morbidity.

### Cell lines

Baby hamster kidney cells (BHK-21; ATCC CCL-10) and murine C3H/An connective tissue L929 cells (ATCC CCL-1) were maintained in RPMI-1640 supplemented with 10% heat-inactivated donor calf serum (DCS; Gibco) and 10% tryptose phosphate broth (Moltox). African green monkey Vero cells were obtained from ATCC (CCL-81) and maintained in Dulbecco’s modified Eagle’s medium (DMEM) supplemented with 10% heat-inactivated fetal bovine serum (FBS). All media contained 100 units/ml penicillin, 0.5 mg/ml streptomycin, and 2 mM L-glutamine.

### Construction of LAV candidates

The cDNA clone for the WT EEEV strain FL93-939 was generously provided by Scott Weaver (University of Texas Medical Branch, Galveston) [54]. The LAV candidates containing mutations in the 5’ UTR (nucleotide mutations: G4A and G6A), capsid protein (deletion of amino acids 65–69), E2 protein (amino acid mutations: K71A, K74A, and K77A; 71–77) [33] and 3’ UTR (deletion of nucleotides 11337–11596) [35] were created using the Quick Change II XL mutagenesis kit (Stratagene). The following primers were used; 5’U4&6-S: CTA ATA CGA CTC ACT ATA GAT AAG ATA CGG TGT AGA GGC AAC CAC CCT ATT TC, 5’U4&6-AS: GAA ATA GGG TGG TTG CCT CTA CAC CGT ATC TTA TCT ATA GTG AGT CGT ATT AG; C65-69-S: CCA ACC CTC CAG CAG GAC CGA AGC CTG CGC CCA AGC CTA; C65-69-AS: TAG GCT TGG GCG CAG GCT TCG GTC CTG CTG GAG GGT TGG; E71-77-S: CCT ACA TGA GTT TCA TGA ACG GCG CAA CGC AGG CAT CAA TAG CGA TCG ACA ACC; E71-77-AS: GCC GTT CAT GAA ACT CAT GTA GGC CAA ATC GAC; 3’U11337-S; GAC ATT AAC ATC TTG TCA ACC GGC AGC GCA TAA TGC TGT CTT TTA TAT C; 3’U11337-AS: GAT ATA AAA GAC AGC ATT ATG CGC TGC CGG TTG ACA AGA TGT TAA TGT C. Fragment swapping strategies were also used for constructing the different combinations of LAVs using the restriction sites (Mlu I, EcoR I, and Not I). All of the LAV candidates were verified by DNA sequencing. Viruses containing all combinations of the four mutant loci were created with the exception of a triple mutant with a wild type 5’ UTR.

### Generation of LAV vaccine stocks

LAV vaccine RNAs were generated using Not I linearized cDNA to make capped, *in vitro* transcribed RNA (mMessage mMachine, Ambion). The RNA was electroporated into BHK-21 cells, and the supernatants were harvested 16–20 hours after electroporation. The supernatant was clarified by centrifugation and stored at -80°C in single use aliquots. Virus titers were determined by a standard plaque assay on BHK-21 cells. To quantify the number of genomes in each LAV stock, 20 U RNase ONE (Promega) was added to 200 μl virus stock (60 min at 37°C) to eliminate free RNA. After incubation, virus supernatant was added to Tri-reagent and frozen at -80°C. Polyacryl carrier was added to each sample, and RNA was isolated according to protocol provided by manufacturer. cDNA was reverse transcribed from 100ng of RNA as previously described [55] using T7-FL93-nsP2-AS: GCG TAA TAC GAC TCA CTA TAT GAC AAC CAA CGA GTG TGG G. Quantitative determination of the number of genomic equivalents (GE) in each LAV stock was performed using SYBR green on a MiniOpticom thermal cycler (Bio-Rad) and previously described conditions [55] with the primers FL93-nsP2-S: AGA GTG GCT GAC GTT CGC AC, and T7: GCG TAA TAC GAC TCA CTA TA to quantify positive-strand RNA. The EEEV GE standard curve was based on 10 fold dilutions of *in vitro* transcribed EEEV replicon RNA [47].

### Sequencing of virus stocks

To sequence the 5’UTR mutation, viral RNA stocks prepared as described above were decapped using RNA 5’ Pyrophosphohydrolase (RppH; New England BioLabs) according to manufacturers’ guidelines. Viral RNA (100 ng) was incubated with RppH in NEB Buffer 2 for 1 h at 37°C followed by addition of 500mM EDTA and heat inactivation at 65°C for 5 min. Viral RNA was cleaned using RNeasy MinElute Cleanup Kit (Qiagen) according to manufacturer’s guidelines and eluted with 14 μl of RNase-free water. 5’ and 3’ ends of the viral RNA were then ligated together using 10mM ATP, 50% PEG8000, 40 units (U)/μl of RNase Inhibitor and 10 U of T4 RNA ligase 1 (New England Biolabs) for 16 h at 16°C. Reverse transcription (RT) of the ligated RNA was performed using SuperScript IV reverse transcriptase (Thermo Fisher Scientific) and a random hexamer primer according to manufacturer’s guidelines. The random hexamer primer was first annealed to the ligated RNA by heating at 65°C for 5 min in the presence of 10mM dNTPs followed by incubation on ice for 5 min. Reverse transcription (RT) was preformed using the following conditions: 23°C for 10 min, 50°C for 30 min, and 80°C for 10 min. cDNA amplification was performed using GoTaq polymerase (Promega) and the following conditions: 95°C for 2 min, then amplification for 40 cycles (denaturing: 95°C for 35 sec, annealing: 55°C for 30 sec, extension: 65°C for 1 min). A lower amplification temperature was used due to the presence of the poly A tail in the ligated viral RNA [56]. RT for sequencing of the other virulence loci was performed using Superscript IV VILO (ThermoFisher Scientist) according to manusfacturer’s guidelines using the anti-sense (AS) primers described below for C65-69 and E71-77 and Oligo(dT) for 3’U11337. PCR amplification was performed as described above. The PCR product from all reactions was excised from a 2% agarose cell using Promega Wizard SV Gel and PCR cleanup system. The PCR product was sequenced at the University of Pittsburgh Genomics Research Core. The following primers were used for PCR amplification and sequencing: 5’U4&6: EEEV 3’U-11208-11228-S: CCG CCA CCG CGT GGT CGT GGC, EEEVnsp1-AS: TGA CTT GAC GAA TGG GCT GTC TGC GT; C65-69: S- CCA TAA CCC TCT ACG GCT GAC CT, AS- CTG TAA CCG TGT CCC CTG GT, E71-77: S- AGG AGA ACC AGG AGA GAT TTG GA, AS- GCA CGC TTG TGA GTG TAA C; 3’U11337: EEEV 3’U-11208-11228-S: CCG CCA CCG CGT GGT CGT GGC, EEEV-T7-CSE AS- TAA TAC GAC TCA CTA TAG GGC GTA TGG AAA AAA TTA ATA TGA TTT TGT AAA TTG ATA TAA AAG ACA GC.

### Virus growth curve

Virus growth curves were performed as described previously with some modifications [47]. Vero cells were infected in triplicated in 24-well plates with equal genomes of each LAV stock corresponding to a multiplicity of infection (MOI) of 1 pfu per cell of WT EEEV. Supernatant was collected at time zero and indicated time points for titration by plaque assay on BHK-21 cells.

### Primary mouse infection and tissue harvest

Outbred 5-6-week-old female CD-1 mice (Charles River) were infected subcutaneously (sc) in each footpad or intracerebrally (ic) with equal genomes of the LAV vaccine stocks corresponding to 10^3^ pfu of WT EEEV (1.5 x 10^5^ genomic equivalents) in OptiMEM (Gibco). Mice were monitored twice daily for morbidity and mortality. Serum was collected via the submandibular vein at indicated time points and stored at -80°C until use. For virus challenge studies, mice were aged for 21 days, and bled on day 21 prior to challenge. For tissue harvest, popliteal lymph nodes (PLN) were harvested at 24 hours post infection (hpi) and placed in 100 μl PBS containing 1% DBS for virus titration.

### Interferon (IFN-α/β) bioassays

Biologically active serum IFN-α/β collected at 24 hpi was measured using a standard biological assay on L929 cells as described previously [47,57]. The IFN-α/β concentration in sera samples was set as the dilution of sample required for 50% protection from cytopathic effect compared to protection conferred by an IFN standard. To generate the IFN standard, murine IFN-α or IFN-β sequences were cloned into a previously described Sindbis virus replicon [58]. Capped, *in vitro* transcribed IFN-α or IFN-β encoded replicon RNA was electroporated into BHK cells and incubated overnight at 37°C. The next day, the supernatant was initially clarified by centrifugation (4000 rpm for 30 min at 4°C) followed by ultracentrifugation at 24,000 rpm for 6 h at 4°C. The supernatant was then acidified to pH = 2.0 with 0.02 N H_2_SO_4_ and incubated overnight at 4°C. The supernatant was neutralized to pH = 7.0 with 0.2N NaOH and concentrated using Amicon Ultra-4 <10mw centrifugal filter units (EMD Millipore). Concentration of mouse IFN-α and IFN-β was determined using the IFN-α/β bioassay and known IFN standards.

### Virus challenge experiments

For sc challenge, on day 22 after primary infection with the LAV candidates, mice were infected sc in both footpads with either 1 x 10^5^ pfu or 1 x 10^4^ pfu of 20% sucrose purified WT EEEV FL93 expressing nanoLuciferase (nLuc) as a self-cleavable protein (TaV) [48]. Aerosol challenge experiments were performed as previously described [59,60]. Briefly, mice were challenged with high doses of 20% sucrose-purified WT EEEV FL93-nLuc TaV using the AeroMP exposure system (Biaera Technologies, Hagerstown, MD) inside a class II biological safety cabinet and either an Aeroneb nebulizer (Aerogen) or 3-jet Collison nebulizer (CH Technologies). All mice were monitored twice daily for morbidity and mortality.

### *In vivo* imaging

On day 4 (aerosol) or day 6 (sc) post challenge, mice were injected with 10 μg of Nano-Glo substrate sc in 500 μl PBS as previously described [59]. Four min after substrate injection, the mice were imaged using the IVIS Spectrum CT Instrument (PerkinElmer) using the autoexposure setting. The total flux (photons per second) in the head region was calculated for each animal using Living Image Software 4.5.1 with all images set to the same scale. Images of representative animals are shown from each LAV vaccine candidate.

### Plaque reduction neutralization titer assay (PRNT)

A chimeric SINV (TR339) encoding the EEEV FL93 structural proteins was generated from *in vitro* transcribed RNA in BHK cells as previously described [50]. Serum collected on D21 from mice immunized with the LAV candidates was heat inactivated at 56°C for 30 min. The serum was serially diluted (2-fold dilutions) and incubated with ~100 pfu of SINV-EEEV for 1 h at 37°C. Anti-EEEV ascites serum (ATCC) was used as a positive control. After incubation, Vero cells were infected in 6 well plates for 1 h at 37°C in a plaque assay. After overlay with agarose immunodiffusion grade (MP Biomedicals), plates were incubated for 2 days followed by overlay with neutral red for at least 6 h to count plaques. Percent neutralization was calculated based on the number of plaques in each serum dilution compared to the number of plaques in non-antibody treated control wells. A best fit non-linear curve was used to calculate the 80 percent reduction dilution (GraphPad Prism).

### Luminex cytokine bead array

CD-1 mice were infected as described above and serum was collected at 24 hpi infection stored at -80°C until use. A mouse 26-plex ProcartaPlex immunoassay (ThermoFisher Scientific) and a Bio-Plex Pro II array washer were used according to manufacturer’s guidelines and 25 μl of serum. Samples were run on a Bio-Rad Bio-Plex II suspension array system in BSL-3 containment. Background values were subtracted from calculated cytokine concentrations.

### Intracellular cytokine staining

C57BL6 mice (6 weeks) were immunized with equal genomes of the triple and quadruple LAVs (1.5 x 10^5^ genomic equivalents) in both rear footpads. On day 6, spleens were harvested, and an intracellular cytokine staining was performed as previously described [61]. Splenocytes were stimulated with 1μM of an EEEV-specific nsP2 peptide (RSFRFSRV, >95% purity GenScript) (D.W. Trobaugh and W.B. Klimstra manuscript in preparation) for 5 hr in the presence of brefeldin A (GolgiPlug, BD Biosciences). Cells were washed with PBS and stained with GhostDye UV450 (Tonbo Biosciences). Next, cells were incubated with 1/200 dilution of anti-CD16/32 for 15 min at 4°C followed by surface staining with 1/100 dilution anti-CD3 PerCP-Cy5.5 (145-2C11) and anti-CD8 APC-Cy7 (53–6.7) for 20 min. After permeabilization with BD CytoFix/CytoPerm, cells were incubated with 1/100 dilution of anti-IFN-γ FITC for 20 min 4°C. Cells were then fixed with 4% PFA overnight. Data was collected on a BD LSR II and analyzed with FloJo software (TreeStar). All antibodies were purchased from Tonbo Biosciences unless specified.

### Statistical analysis

All statistical analysis was performed using GraphPad Prism software. Statistical significance for survival curves was determined by Mantel-Cox log rank test. In general, WT EEEV was compared to the single mutants, the single mutants were compared to the double mutants incorporating the single mutations, the double mutants were compared to the triple mutants incorporating the double mutations, and the triple mutants were compared to the quadrupole mutant. Comparisons indicated in figure legends were determined by one-way analysis of variance with Turkey’s multiple-comparison test of log-transformed data or two-way analysis of variance with multiple comparisons using the Bonferroni method.

## Supporting information

S1 Fig Mouse weight loss after subcutaneous EEEV challenge. Mice were immunized with equal genomes of each indicated LAV in both rear footpads. On day 22, mice were challenged subcutaneously with WT EEEV-nLuc in the left footpad with 10^4^−10^5^ pfu. Mice were weighed daily and percent change in weight was calculated from the initial weight on day 0 of experiment. X-axis represents days post challenge with 0 being day 22 of experiment. Each line represents an individual mouse from 2 independent experiments. Red line indicates mice that did not survive challenge.
(TIF)

S2 Fig Mouse weight loss after aerosol EEEV challenge. Mice were immunized with equal genomes of each indicated LAV in both rear footpads. On day 22, mice were challenged with 100 LD_50_ of EEEV expressing nLuc. Mice were weighed daily and percent change in weight was calculated from the initial weight on day 0 of experiment. X-axis represents days post challenge with 0 being day 22 of experiment. Each line represents an individual mouse from 2–3 independent experiments. Red line indicates mice that did not survive challenge.
(TIF)

S3 Fig Mouse weight loss after high dose aerosol EEEV challenge. Mice were immunized with equal genomes of each indicated LAV in both rear footpads. On day 22, mice were challenged with >1000 LD_50_ of EEEV expressing nLuc. Mice were weighed daily and percent change in weight was calculated from the initial weight on day 0 of experiment Mice were weighed daily and percent change in weight was calculated from the weight on day 0 of experiment. X-axis represents days post challenge with 0 being day 22 of experiment. Each line represents an individual mouse and red line indicates mice that did not survive challenge.
(TIF)
