# Supplementary figures and images for: Rational design of a live-attenuated eastern equine encephalitis virus vaccine through informed mutation of virulence determinants
Source: PLoS Pathog. 2019 Feb 11;15(2):e1007584. doi: 10.1371/journal.ppat.1007584 (PMC6386422; doi:10.1371/journal.ppat.1007584)

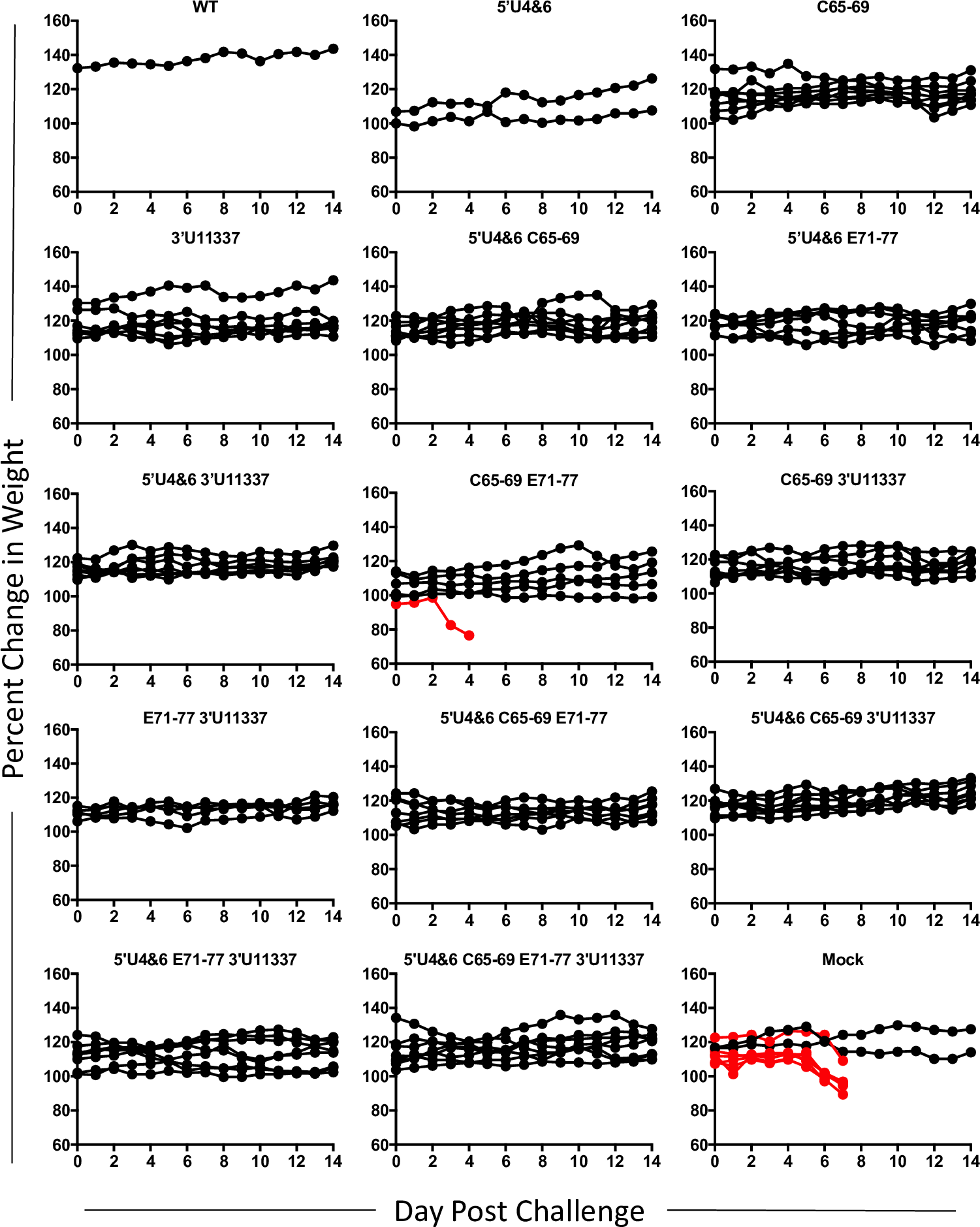

Supplement: S1 Fig — Mice were immunized with equal genomes of each indicated LAV in both rear footpads. On day 22, mice were challenged subcutaneously with WT EEEV-nLuc in the left footpad with 104−105 pfu. Mice were weighed daily and percent change in weight was calculated from the initial weight on day 0 of experiment. X-axis represents days post challenge with 0 being day 22 of experiment. Each line represents an individual mouse from 2 independent experiments. Red line indicates mice that did not survive challenge. (TIF) [file ppat.1007584.s001.tif]

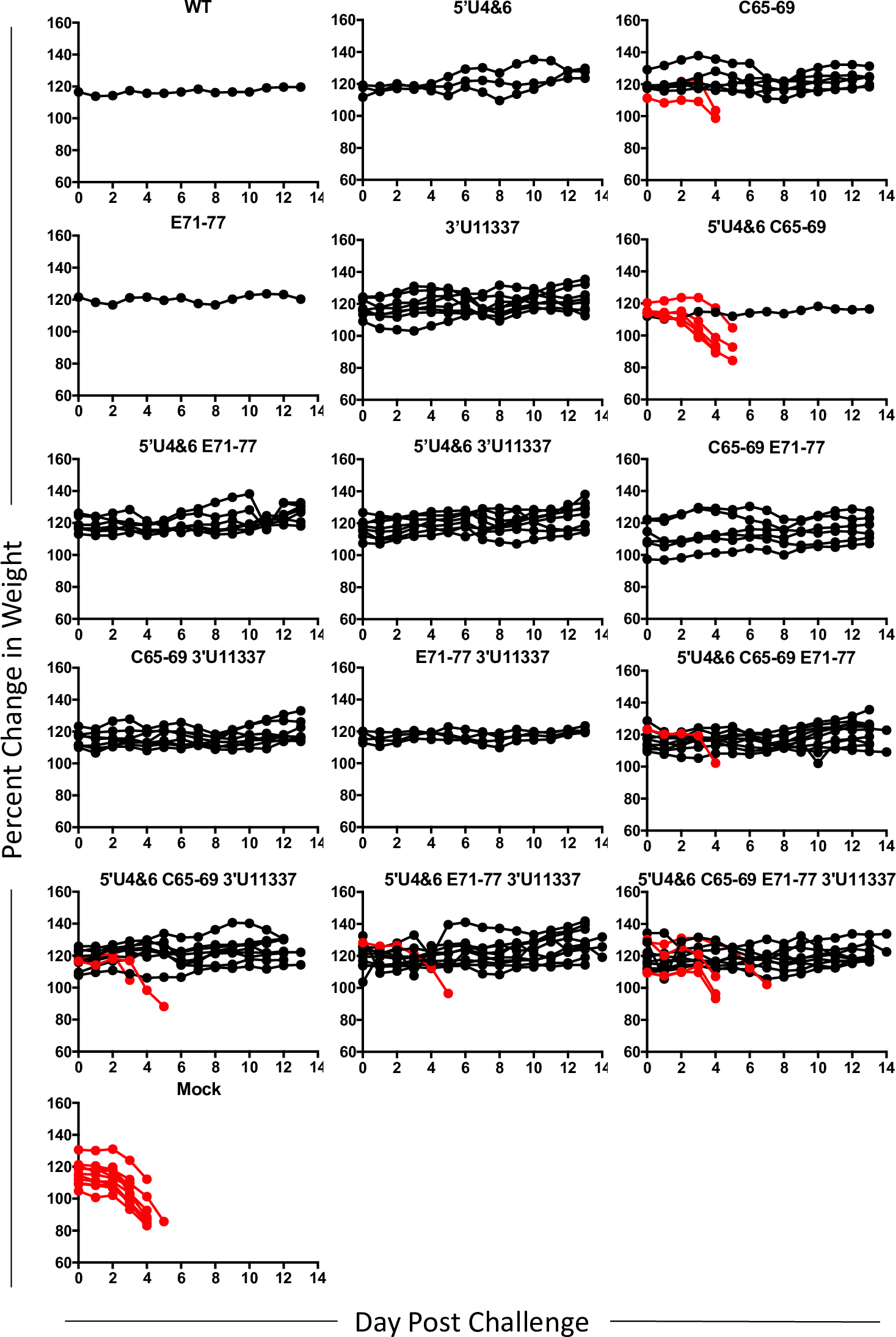

Supplement: S2 Fig — Mice were immunized with equal genomes of each indicated LAV in both rear footpads. On day 22, mice were challenged with 100 LD50 of EEEV expressing nLuc. Mice were weighed daily and percent change in weight was calculated from the initial weight on day 0 of experiment. X-axis represents days post challenge with 0 being day 22 of experiment. Each line represents an individual mouse from 2–3 independent experiments. Red line indicates mice that did not survive challenge. (TIF) [file ppat.1007584.s002.tif]

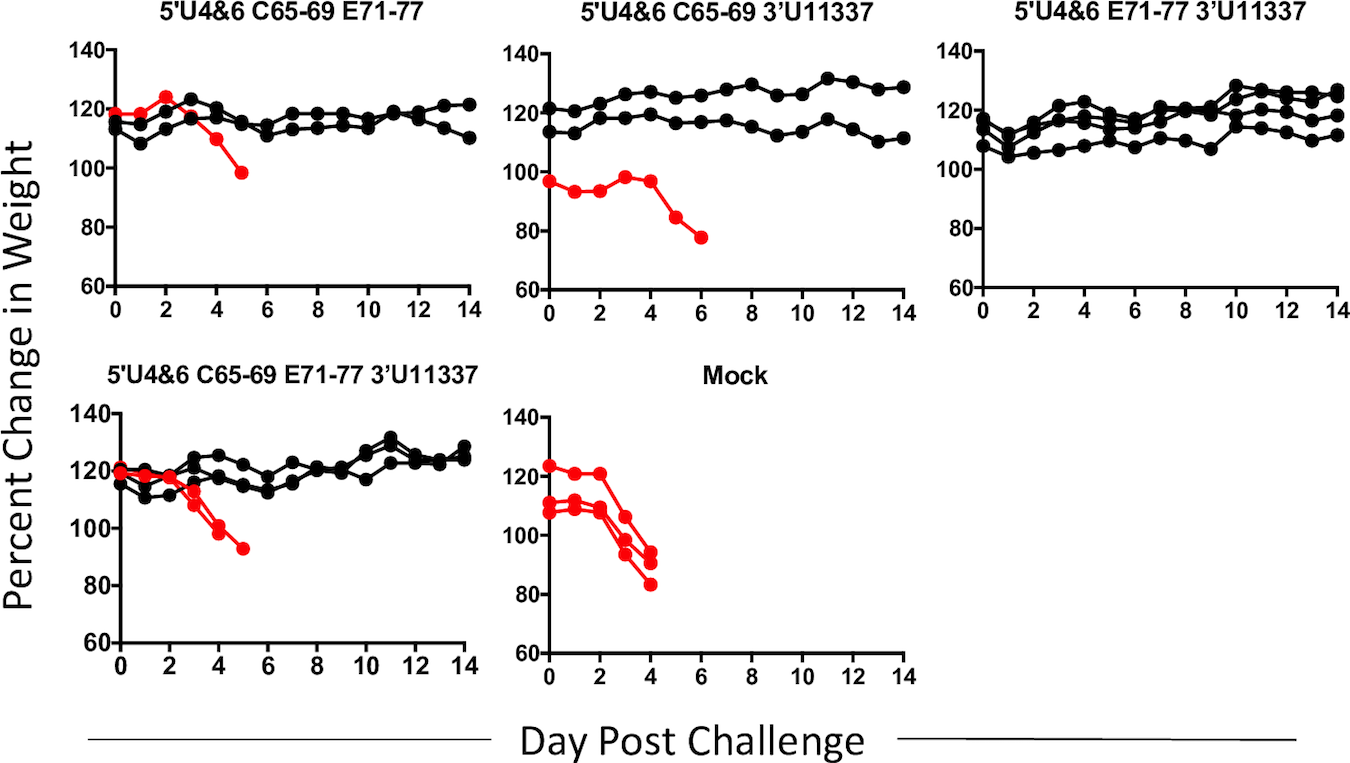

Supplement: S3 Fig — Mice were immunized with equal genomes of each indicated LAV in both rear footpads. On day 22, mice were challenged with >1000 LD50 of EEEV expressing nLuc. Mice were weighed daily and percent change in weight was calculated from the initial weight on day 0 of experiment Mice were weighed daily and percent change in weight was calculated from the weight on day 0 of experiment. X-axis represents days post challenge with 0 being day 22 of experiment. Each line represents an individual mouse and red line indicates mice that did not survive challenge. (TIF) [file ppat.1007584.s003.tif]
